# Deep learning-based brain transcriptomic signatures associated with the neuropathological and clinical severity of Alzheimer’s disease

**DOI:** 10.1093/braincomms/fcab293

**Published:** 2021-12-14

**Authors:** Qi Wang, Kewei Chen, Yi Su, Eric M. Reiman, Joel T. Dudley, Benjamin Readhead

**Affiliations:** ASU-Banner Neurodegenerative Disease Research Center, Arizona State University, Tempe, AZ 85281, USA; Banner Alzheimer’s Institute, Phoenix, AZ 85006, USA; Banner Alzheimer’s Institute, Phoenix, AZ 85006, USA; ASU-Banner Neurodegenerative Disease Research Center, Arizona State University, Tempe, AZ 85281, USA; Banner Alzheimer’s Institute, Phoenix, AZ 85006, USA; ASU-Banner Neurodegenerative Disease Research Center, Arizona State University, Tempe, AZ 85281, USA; Icahn School of Medicine at Mount Sinai, New York, NY 10029, USA; ASU-Banner Neurodegenerative Disease Research Center, Arizona State University, Tempe, AZ 85281, USA

**Keywords:** AMP-AD, transcriptome, deep learning, pseudo-temporal trajectory, biomarker

## Abstract

Brain tissue gene expression from donors with and without Alzheimer’s disease has been used to help inform the molecular changes associated with the development and potential treatment of this disorder. Here, we use a deep learning method to analyse RNA-seq data from 1114 brain donors from the Accelerating Medicines Project for Alzheimer’s Disease consortium to characterize post-mortem brain transcriptome signatures associated with amyloid-β plaque, tau neurofibrillary tangles and clinical severity in multiple Alzheimer’s disease dementia populations. Starting from the cross-sectional data in the Religious Orders Study and Memory and Aging Project cohort (*n* = 634), a deep learning framework was built to obtain a trajectory that mirrors Alzheimer’s disease progression. A severity index was defined to quantitatively measure the progression based on the trajectory. Network analysis was then carried out to identify key gene (index gene) modules present in the model underlying the progression. Within this data set, severity indexes were found to be very closely correlated with all Alzheimer’s disease neuropathology biomarkers (*R* ∼ 0.5, *P* < 1e−11) and global cognitive function (*R* = −0.68, *P* < 2.2e−16). We then applied the model to additional transcriptomic data sets from different brain regions (MAYO, *n* = 266; Mount Sinai Brain Bank, *n* = 214), and observed that the model remained significantly predictive (*P* < 1e−3) of neuropathology and clinical severity. The index genes that significantly contributed to the model were integrated with Alzheimer’s disease co-expression regulatory networks, resolving four discrete gene modules that are implicated in vascular and metabolic dysfunction in different cell types, respectively. Our work demonstrates the generalizability of this signature to frontal and temporal cortex measurements and additional brain donors with Alzheimer’s disease, other age-related neurological disorders and controls, and revealed that the transcriptomic network modules contribute to neuropathological and clinical disease severity. This study illustrates the promise of using deep learning methods to analyse heterogeneous omics data and discover potentially targetable molecular networks that can inform the development, treatment and prevention of neurodegenerative diseases like Alzheimer’s disease.

## Teaser

A pseudo-temporal trajectory was derived for Alzheimer’s disease progression from brain transcriptome in a large clinical cohort. The numeric index of the progression is highly predictive of neuropathology and clinical severity in independent data sets. Network analysis identified genes enriched in different cell types implicated for Alzheimer’s disease’s progression.

## Graphical Abstract

**Graphical Abstract:**
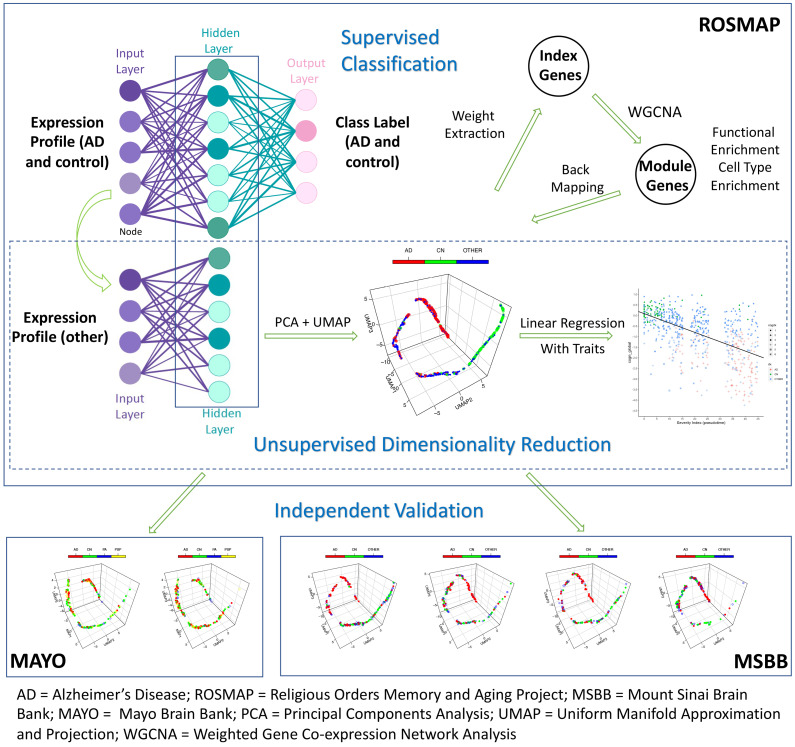

## Introduction

As the age of the global population advances, dementia, with late-onset Alzheimer’s disease (LOAD) as the most prevalent form, has become a formidable public health threat. Despite numerous recent scientific advances in illuminating the pathophysiology of LOAD, no disease-modifying treatments are currently available. This fact underscores the complicated molecular aetiology driving the disease and the urgent need to broaden our search for effective therapeutics beyond the conventional amyloid cascade hypothesis.^1^

For a highly heterogeneous, multifactorial disease such as LOAD, integrated and large-scale genomic data analyses have been carried out to disentangle and capture the diverse gene regulatory interactions.^2^ Most of these studies focus on the exploration of the molecular mechanism of Alzheimer’s disease pathology by employing a case–control study design or modelling it as discrete stages, usually excluding the study subjects with other dementia pathologies, or omitting the mild cognitive impairment stage and its role in the disease progression, even in the studies aiming to reveal the transcriptional dysregulation involving the progression of Alzheimer’s disease.^3^ Difficulties in sampling brain tissue throughout life coupled with globally limited access to diagnostic neuroimaging necessitates that a definitive diagnosis of Alzheimer’s disease is only made following post-mortem neuropathological assessment. This further exacerbates the challenge we face in studying LOAD or dementia in general as a continuous spectrum to find novel biomarkers and drug targets. Recent efforts have begun to model Alzheimer’s disease progression as a continuous trajectory using cross-sectional transcriptomic data,^4,5^ by leveraging the methods developed in single-cell genomics^6,7^ and machine learning.^8^ Iturria-Medina *et al.*^4^ adopted an unsupervised machine learning algorithm applied to gene expression microarray data and discovered a contrastive trajectory in multiple cohorts, respectively. The trajectory has been demonstrated to strongly predict neuropathological severity in Alzheimer’s disease in each data set. Mukherjee *et al.*^5^ applied a manifold-learning method to RNA-seq data to define ordering across samples based on gene expression similarity and estimate the disease pseudotime for each sample. Disease pseudotime was strongly correlated with the burden of Aβ, tau and cognitive dementia within subjects with LOAD. Although these unsupervised machine learning methods have been shown to be highly predictive for well-known pathological biomarkers within a data set, it would be desirable to have a generalized, universally predictive model for Alzheimer’s disease neuropathology and cognitive impairment across distinct cohorts and brain tissues, which helps decipher common Alzheimer’s disease aetiology at the molecular level. In addition, their broader application in peripheral tissues to identify novel biomarkers would greatly facilitate early diagnosis and progression monitoring of Alzheimer’s disease.

Deep learning methodologies are a rapidly evolving class of machine learning algorithms that have demonstrated superior performance over traditional machine learning approaches in identifying intricate structures in complex high-dimensional data, across diverse domains including computer vision, pattern recognition and bioinformatics.^9^ Specific to genomic data, it has been demonstrated that ‘big data’ in many human diseases can be exploited by deep learning methods for early detection,^10^ disease classification^11,12^ and biomarker identification,^8,13^ mostly in the cancer research field. More recent efforts have begun to apply similar methods towards research questions within the neuroscience research field,^14^ including the study of neurodegenerative disease,^15^ though the potential for these methods to contribute to novel insights in Alzheimer’s disease research remains underexplored.

In this work, we are leveraging the multidimensional, well-characterized and high-quality genomic, neuropathological and clinical data from the Accelerating Medicines Project for Alzheimer’s Disease (AMP-AD) programme^16^ and applying the latest deep learning framework to identify pseudo-temporal trajectories in transcriptomic space and the underlying gene signatures for Alzheimer’s disease progression. As a major component of the AMP-AD programme, the Target Discovery and Preclinical Validation Project brings together different organizations to collect and analyse multidimensional molecular data (genomic, transcriptomic, epigenomic, proteomic) from more than 2000 human brains and peripheral tissues from multiple Alzheimer’s disease cohorts.^17^ Using the RNA-seq data from dorsolateral prefrontal cortex (DLPFC) region in the Religious Orders Study and Memory and Aging Project (ROSMAP) cohort,^18,19^ we first trained a deep learning model to perform supervised classification between the two termini of the disease continuum (Alzheimer’s disease and control diagnosis group). The goal is to achieve the maximum separation of neuropathologically confirmed cases and controls. The model was subsequently applied to all the subjects within the cohort and the intermediate layer of the obtained manifold for all subjects was further dimensionality reduced by Uniform Manifold Approximation and Projection (UMAP)^20^ to obtain a trajectory in three-dimensional (3D) space for Alzheimer’s disease progression. We then derived an index to assess the stage of the progression, namely the severity index (SI) along the trajectory. We observed that the SI was significantly correlated with all the neuropathological biomarkers and achieved excellent model metrics aligned with the global cognitive function scores. When the deep learning model trained on the ROSMAP cohort was applied to two independent AMP-AD data sets, the MAYO RNA-seq study cohort^21^ and The Mount Sinai Brain Bank (MSBB) study cohort,^22^ similar trajectories and sample distribution following a generalized pattern were observed and the estimated SI values remain to be strongly correlated to pathological biomarkers and clinical severity. The model identified 593 genes (‘index genes’) playing significant roles for the severity of Alzheimer’s disease-related neuropathology and cognitive impairment in the disease continuum. Network analysis suggests that these genes are clustered in six gene co-expression modules, four of which are strongly associated with neuropathology and clinical severity. One of the four modules shows especially high correlation with all the neuropathological biomarkers and clinical cognitive functions and the genes are associated with metabolic and vascular dysfunction in oligodendrocytes. The other three modules are also found to be associated with the pathological and clinical severity significantly in neurons, astrocytes and endothelial cells, respectively. Our results collectively demonstrate that a deep learning approach can reveal novel genomic information from complex, high-dimensional gene expression data in a manner that can elucidate the molecular mechanisms of Alzheimer’s disease. The model can be readily applied to additional gene expression data sets to predict Alzheimer’s disease severity, thus indicating its potentially broad utility for Alzheimer’s disease diagnosis and staging. The approach also provides a general framework for studying multi-omics data to capture underlying molecular signatures towards novel biomarkers and drug targets of neurogenerative diseases.

## Materials and methods

### RNA-seq data sets from AMP-AD consortium

All the RNA-seq data were obtained from the AMP-AD data portal through Synapse (https://www.synapse.org/). Demographic information for each of the cohort (ROSMAP, MAYO and MSBB) sampled in the RNA-seq study is reported in Supplementary Table 1. The processed, normalized data were obtained for each cohort, respectively, from the harmonized, uniformly processed RNA-seq data set across the three largest AMP-AD contributed studies (syn17115987). In the ROSMAP cohort, all the brain tissue samples were collected from DLPFC (*n* = 639, syn8456629).^18,19^ In Mayo RNA-seq study (syn8466812), brain tissue samples were collected from cerebellum (CER, *n* = 275) and temporal cortex (TCX, *n* = 276).^21^ The MSBB study (syn8484987) has 1096 samples from the Mount Sinai/JJ Peters VA Medical Center Brain Bank, which were sequenced from 315 subjects from four brain regions including the frontal pole [FP, Brodmann area (BM) 10], inferior frontal gyrus (IFG, BM 44), superior temporal gyrus (STG, BM 22) and parahippocampal gyrus (PHG, BM 36), respectively.^22^ The harmonized processing of each study from three cohorts was previously performed using a consensus set of tools with only library type-specific parameters varying between pipelines (https://github.com/Sage-Bionetworks/ampad-DiffExp).^23^ The log counts per million reads (CPM) values from each data set were used in all the subsequent analyses.

### Phenotypic data

All the clinical and pathological data for the ROSMAP cohort were obtained from the Rush Alzheimer’s Disease Center Research Resource Sharing Hub (https://www.radc.rush.edu/home.htm), upon approval of data-usage agreement. The following phenotypical measurements were used in the study: cogdx = final consensus cognitive diagnosis; age_death = age at death; educ = years of education; msex = sex; race7 = racial group; apoe4 = apoe4 allele count; PMI = post-mortem interval; r_pd = clinical Parkinson’s disease; r_stroke = stroke diagnosis; dlbdx = pathologic diagnosis of Lewy body diseases; hspath_typ = hippocampal sclerosis; arteriol_scler = arteriolosclerosis; braaksc = Braak stage; ceradsc = Consortium to Establish a Registry for Alzheimer’s Disease (CERAD) score; gpath = global Alzheimer’s disease pathology burden; niareagansc = National Institute on Aging-Reagan diagnosis of Alzheimer’s disease; amyloid = overall amyloid level; plaq_d = diffuse plaque burden; plaq_n = neuritic plaque burden; nft = neurofibrillary tangle burden; tangles = tangle density; cogn_global = global cognitive function. All the clinical diagnosis data were from the last visit, except for cogn_global, which was from the last available test. Their detailed definitions, together with possible values are reported in Supplementary Table 2. Among them, cogdx, braaksc and ceradsc values were used to define the class label for Alzheimer’s disease, control (CN) and OTHER groups (see below, methods for deep learning).

For MAYO and MSBB cohorts, subject clinical and pathological data were obtained from Synapse (syn3817650 for Mayo TCX samples, syn5223705 for Mayo cerebellum samples and syn6101474 for all the MSBB samples). For MAYO cohort, the following phenotypical data were used in the linear regression: age_death = age at death; gender = sex; apoe4 = apoe4 allele count; RIN = RNA integrity number; PMI = post-mortem interval; Braak = Braak stage; Thal = Thal amyloid stage. For MSBB cohort, the following phenotypical data were used in the linear regression: age = age at death; sex = sex; race = racial group; apoe4 = apoe4 allele count; RIN = RNA integrity number; PMI = post-mortem interval; Braak = Braak stage; PlaqueMean = mean plaque burden; CDR = clinical dementia rating; CERAD = CERAD score. The original CERAD score in the MSBB cohort was defined as: 1, Normal; 2, Definite Alzheimer’s disease; 3, Probable Alzheimer’s disease; 4, Possible Alzheimer’s disease. They were recoded to be semi-quantitative as follows: 1, Definite Alzheimer’s disease; 2, Probable Alzheimer’s disease; 3, Possible Alzheimer’s disease; and 4, Normal, to be consistent with the notion used in the ROSMAP cohort.

### Deep learning of the transcriptome from DLPFC tissues in ROSMAP cohort

**Figure 1:**
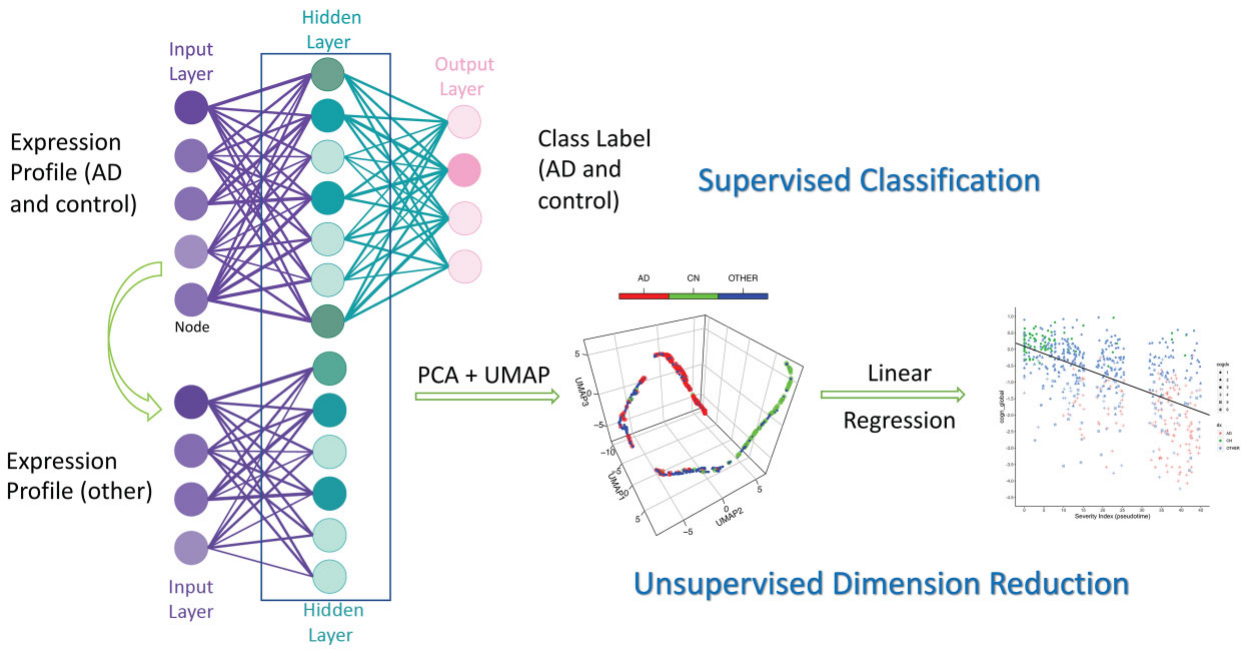
**The deep learning framework employed in this work**. Using the gene expression profiles from Alzheimer’s disease and control subjects and their diagnosis class as the input for supervised classification, the model was trained by a three-layer neural network. Then the response function from input layer to the hidden layer was applied to the profiles from the whole cohort. The resulting manifold was subject to unsupervised dimension reduction (PCA + UMAP) to obtain the pseudo-temporal trajectory and SI. SI was linearly correlated with phenotypic data for evaluation.

The whole machine learning framework consists of two major components, supervised classification (deep learning) and unsupervised dimension reduction. The deep learning method was built wholly based on the approach implemented in the previous implementation DeepType (https://github.com/runpuchen/DeepType).^11^ The detailed algorithm could be found in the reference. The method has been demonstrated to achieve superior performance on independent data sets and is very robust against label noise in classifying genomic data from complex human diseases such as cancer.^24^ In this work, we incorporated the method into our model (supervised classification) and applied it to the normalized logCPM data from the ROSMAP cohort, which consists of the expression profile of 634 subjects with various Alzheimer’s disease pathology for 15 582 genes, and further applied an unsupervised dimension reduction method to obtain the pseudo-temporary trajectory for Alzheimer’s disease progression. The whole framework is illustrated in Fig. 1.

For the deep learning step, we used neuropathologically confirmed Alzheimer’s disease patients and normal controls, the two termini of the Alzheimer’s disease continuum, to train the model and identify transcriptomics signatures that differentiate the two groups. Interpretation of the diagnosis was as follows:

1. Alzheimer’s disease (156 samples): cogdx = 4, braaksc ≥ 4 and ceradsc ≤ 2;
2. CN (87 samples): cogdx = 1, braaksc ≤ 3 and ceradsc ≥ 3;
3. OTHER (391 samples): all the other samples.

This was consistent with the criteria used in the previous differential expression (DE) analysis (syn8456629).^23^ Genes were first sorted in a descending order by the variance of logCPM values for the whole data set. The deep learning model was first built for the 243 samples from Alzheimer’s disease and control diagnosis groups. Data were randomly partitioned into training and test data sets, containing 80% (195) and 20% (48) of the samples with balanced distribution from each group. The logCPM values in the training set were first converted to *Z*-score, followed by scaling those in the test set to the same scale. A three-layer neural network was trained, with the number of the nodes in the input layer, the intermediate layer and the output layer set to 15 582, 128 and 1, respectively. In DeepType, the Adam method^25^ was employed to tune the parameters of the model and a semi-supervised approach was adopted to train three hyper-parameters: the number of clusters *K*, the trade-off parameter *α* and the regularization parameter *λ*. The learning rate was set to 1e−4, the number of training epochs for model initialization and the joint supervised and unsupervised training were set to 1500 and 5000, respectively, and the batch size was set to 256. The model was trained by 5-fold internal cross-validation for the training set and the optimal *K*, *α* and *λ* were determined by the cross-validation to be 2, 2 and 0.004, respectively. Training and validation losses in the training process were tracked to avoid over-fitting.

After the training process was accomplished, a manifold representation of the intermediate layer was obtained for all the 634 samples in the whole cohort by forward pass using the trained network. Prior to that, data were scaled to the *Z*-score using the same mean and standard deviation as the training set. The equation, as implemented in DeepType in MATLAB language, is as follows [Equations (1)–(3)]:

(1) $$\begin{array}{rl} \mathrm{hidden}\_\mathrm{layer} & =\mathrm{sigmoid}(W1^{\prime }\;*\;\mathrm{input}\_\mathrm{data}\\ & \;+\mathrm{repmat}(B1^{\prime },\;[1,\;\mathrm{size}(\mathrm{input}\_\mathrm{data},\;2)])); \end{array}$$

 

(2) hidden_layer=min(hidden_layer,1−1e−9);

 

(3) hidden_layer=max(hidden_layer,1e−9);

where *W*1 is the first-layer weighting matrix and *B*1 is the first-layer bias vector obtained from the model. The hidden layer was bounded between (0, 1). Input_data was the expression matrix with data scaled and sorted in the same order as in the training set.

The resulting representation of the hidden_layer was further dimensionality reduced, first to 50 dimensions, by efficient computation of a truncated principal components analysis (PCA) using an implicitly restarted Lanczos method as implemented in the R package Monocle3,^26^ using the function preprocess_cds, without normalization or scaling. It was further reduced to the first three dimensions, by the UMAP^20^ method as implemented in the R package uwot (https://github.com/jlmelville/uwot). To ensure reproducibility, the following parameters were set: n_components = 3, nn_method = ‘annoy’, n_neighbours = 15L, metric = ‘cosine’, min_dist = 0.1, fast_sgd = F, ret_model = T, with random seed set to 2016.

### Severity index calculation and correlation with phenotypic data in ROSMAP cohort

SI for Alzheimer’s disease progression was derived for each sample, based on the 3D UMAP trajectory obtained earlier, by applying the method of inferring pseudotimes for single-cell transcriptomics from the function ‘slingPseudotime’ as implemented in the R package Slingshot.^27^ SIs were then linearly correlated with all the Alzheimer’s disease clinical and pathological biomarkers individually, including the covariates r_pd, r_stroke, dlbdx, hspath_typ, arteriol_scler, PMI, RIN, apoe4, age_death, educ, msex and race7 (detailed definitions can be found in Supplementary Table 2 and data collection is reported in Tasaki *et al.*^28^), using the following linear regression model:

(4) $$\begin{array}{cc} \mathrm{biomarker}\;∼ & \;\mathrm{SI}+\mathrm{age}\_\mathrm{death}+\mathrm{educ}+\mathrm{msex}+\mathrm{race}\\ & +\mathrm{apoe}4+\mathrm{RIN}+\mathrm{PMI}+r\_\mathrm{pd}+r\_\mathrm{stroke}\\ & +\mathrm{dlbdx}+\mathrm{hspath}\_\mathrm{typ}+\mathrm{arteriol}\_\mathrm{scler} \end{array}$$

The pathological biomarkers and Alzheimer’s disease clinical measures used as dependent variables in the model are braaksc, ceradsc, niareagansc, gpath, amyloid, plaq_d, plaq_n, nft, tangles and cogn_global. All the neuropathological measurements (gpath, amyloid, plaq_d, plaq_n, nft, tangles) were log-transformed in the correlation analysis. All the semi-quantitative and quantitative measurements were treated as numerical; diagnosis of Lewy body diseases, gender and race were treated as categorical. Correlation coefficients were obtained by the ‘lm’ function in R. The proportion of variance explained (PVE) for each predictor was obtained from the incremental sums of squares table by the ‘anova’ function in R on the model, using the above order.

### Applying the deep learning model to external data sets (MAYO, MSBB)

The harmonized, uniformly processed RNA-seq data sets were first sorted by the same gene order as the input data set of ROSMAP. Batch effects were then removed by the ComBat function^29^ in the R package sva.^30^ The input expression matrix subsequently was transformed to *Z*-score by scaling to the training set in the deep learning model. A manifold representation was obtained for all the samples in each cohort by forward pass of the trained network, using Equations (1)–(3) and reduced again to 50 dimensions by PCA. Trajectories were obtained by carrying out the UMAP transformation of the existing embedding model from ROSMAP DLPFC data, by the ‘umap_transform’ function in R package uwot. SI for each sample was again derived from ‘slingPseudotime’ function in Slingshot.^27^ Linear correlation of the SIs with all the pathological and clinical biomarkers was carried out by the ‘lm’ function in R, using other non-Alzheimer’s disease pathology-related variables as covariates when available (age, sex, race, PMI, RIN, apoe4 allele counts), by the following linear regression model:

(5) $$\begin{array}{cc} \mathrm{biomarker}∼ & \mathrm{SI}+\mathrm{age}\_\mathrm{death}+\mathrm{sex}+\mathrm{race}+\mathrm{apoe}4\\ & +\mathrm{RIN}+\mathrm{PMI} \end{array}$$

### Network and cell-type analysis of the significant genes underlying AD progression

The hidden layer of the deep learning model returned a weight vector for each of the 15 582 genes in the input data set. The root sum squares (RSS) of the weight vector for each gene was calculated, normalized to the maximal RSS and taken as the weight for each gene in the deep learning model. The weights from all genes were put into a histogram on a logarithm scale and a cut was made to separate the bimodal distribution. The genes in the higher weight groups were identified as significant genes contributing to Alzheimer’s disease progression (index genes). Unsigned co-expression networks were built for these genes’ expression profiles using the unscaled logCPM values. Network modules were identified using the cutreeDynamic function in the R package WGCNA,^31^ setting the minimum module size to 30. The power of four was chosen using the scale-free topology criterion. Correlation of 0.35 or height cut of 0.35 with deepSplit = 4 was used to merge modules whose genes are highly co-expressed.

Functional enrichment analysis was performed using Metascape,^32^ which uses a hypergeometric test and Benjamini–Hochberg *P*-value correction to identify ontology terms that contain a statistically greater number of genes in common with an input list than expected by chance, using the whole transcriptome as background. Statistically significant enriched terms based on Gene Ontology,^33^ KEGG,^34^ Reactome^35^ and MSigDB^36^ were clustered based on Kappa-statistical similarities among their gene memberships. A 0.3 kappa score was applied as a threshold to identify enriched terms.

Fisher’s exact test was used to test the enrichment of the gene set from each module with the gene sets generated for the ROSMAP samples from the meta-analysis of Alzheimer’s disease co-expression modules,^23^ or other curated Alzheimer’s disease gene sets (Supplementary material). The resulting *P*-values were corrected using the Bonferroni method for multiple test correction. Cell-type enrichments were also done using Fisher’s exact test of gene set to overlap with cell-type-specific gene sets from human reference single-cell RNA-seq data^37^ and the unique marker genes present in the single-cell RNA-seq data (with log_2_FC > 1) from the prefrontal cortical samples of Alzheimer’s disease patients and normal control subjects.^38^

Cell-type marker gene expression signatures along SI were obtained by first smoothing each gene’s expression as a function of SI using a smoothing spline of the degree of freedom = 3. The weighted mean of the marker genes was obtained and normalized to lie in [0, 1]. The smoothed and normalized expression of marker genes for each cell type was plotted as a function of SI.

### Statistical analysis

All the statistical tests were carried out in R version 4.0.0,^39^ except for functional enrichment analysis performed using Metascape, where the *P*-values were directly reported by the programme. The details of the tests were reported in each section of the methods above.

### Data availability

All the data sets from the AMP-AD consortium used in this study are available at the Alzheimer’s disease knowledge portal (https://adknowledgeportal.synapse.org/), with synapse identifiers provided in the text. The machine learning framework, including the trained model, SIs for each cohort and the codes to apply the trained neural network, map to the DLPFC 3D UMAP space and obtain the SI will be uploaded to synapse upon publication of this work. The source code with synapse data withheld is available at https://github.com/qwang178/DeepBrain.

## Results

### Deep learning identified a pseudo-temporal trajectory for AD progression

We designed a three-layer deep learning model to dissect the gene expression data from DLPFC tissues across the Alzheimer’s disease spectrum. This simple scheme consists of inputting the two termini of the spectrum (i.e. pathologically confirmed Alzheimer’s disease and control groups) to obtain a learned representation encoded by the intermediate layer. We set the number of output clusters *K* at 2 throughout the learning process (i.e. we are not interested in any subcluster within the two termini). The regularization parameter *λ* and the trade-off parameter *α* were estimated to be 0.004 and 2, respectively (Supplementary Fig. 1A and B). The training and validation losses in the training process were tracked and no sign of over-fitting was observed (Supplementary Fig. 1C). The training and validation accuracy was observed to be stable at ∼97 and ∼90%, respectively (Supplementary Fig. 1D).

**Figure 2:**
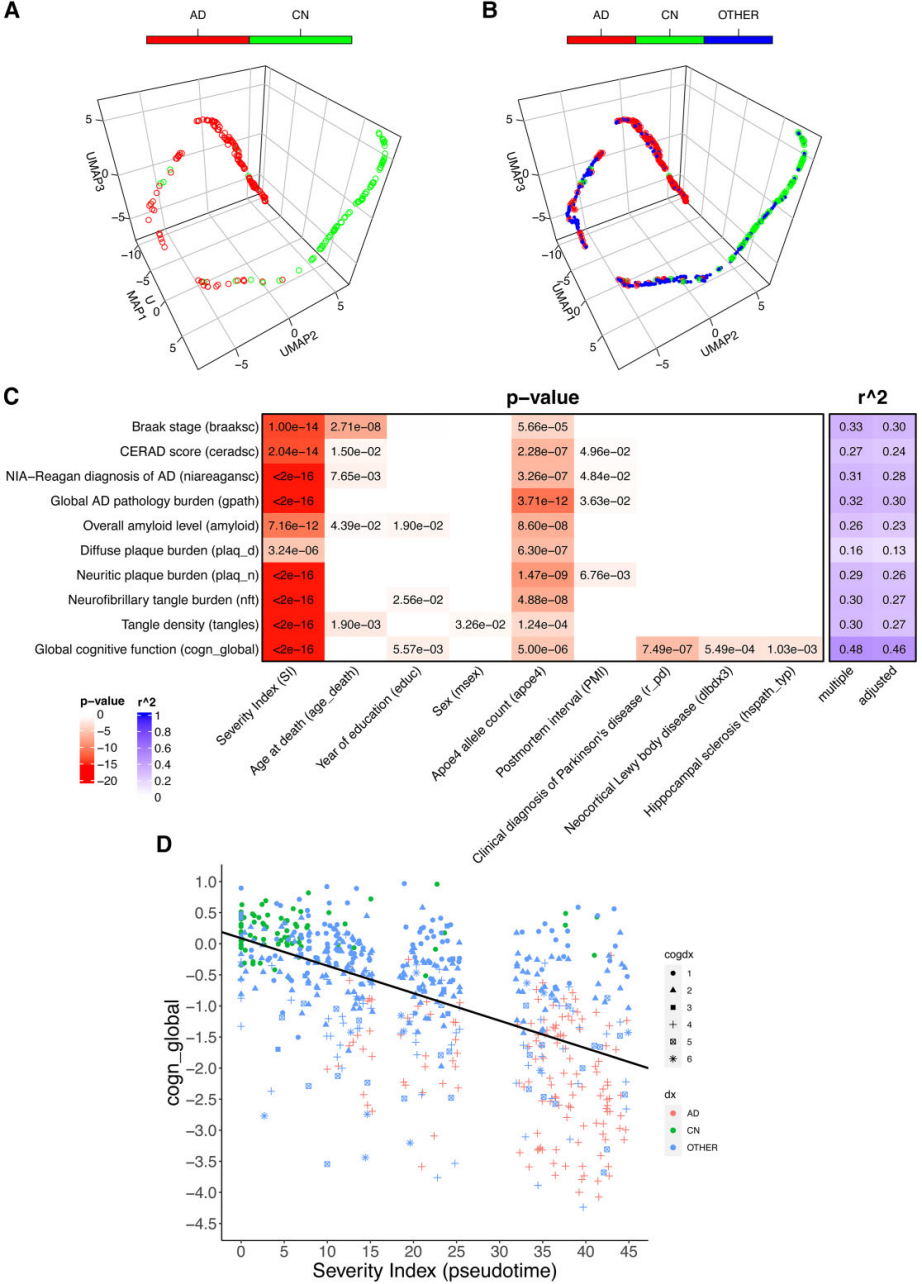
**The pseudo-temporal trajectory from the trained deep learning model for the transcriptome from DLPFC tissues of ROSMAP cohort and the SI correlation with phenotypical data**. Diagnosis class is defined in the main text. (**A**) The trajectory with only Alzheimer’s disease and control (deep learning data set) is shown. (**B**) The trajectory with all subjects (Alzheimer’s disease + CN + OTHER) is shown. (**C**) The model metrics for the linear regression between SI, controlled covariates and all the neuropathological biomarkers and global cognitive function, with *P*-values shown in cells with *P* < 0.05. Only covariates with significant association with at least one biomarker were shown. The descriptions for the definition of each parameter can be found in Supplementary Table 2. Detailed model metrics stratified by diagnosis groups can be found in Supplementary Tables 3 and 4. (**D**) The linear regression plot between SI and global cognitive function. Colour legend for cogdx (final consensus cognitive diagnosis): 1, no cognitive impairment (CI); 2, MCI and no other cause of CI; 3, MCI and another cause of CI; 4, Alzheimer’s disease and NO other cause of CI; 5, Alzheimer’s disease and another cause of CI; 6, other dementia.

After the intermediate layer was mapped into 3D UMAP space, a prominent progressive trajectory, with two distinct clusters at both termini could be observed (Fig. 2A). Mapping of the other samples into the same space clearly indicated a continuous disease spectrum as well as a progression course along the trajectory (Fig. 2B). SI was calculated as the travelling distance along the trajectory by setting the starting point at the control terminus to zero, which reflects the disease progression of the subjects. When correlating with pathological biomarkers, the SI showed strong correlations with all the measurements (*P* ≤ 3.2e−6), with the weakest correlation observed for diffuse plaque, which was still highly significant (*P* = 3.2e−6) (Fig. 2C). In addition, it indicated that APOE4 allele counts also contributed to all the biomarkers with various degrees of significance (*P* = 1.24e−4 to 1.47e−9), confirming it as a major genetic risk determinant for Alzheimer’s disease. Most strikingly, the model explained the greatest amount of variance for global cognitive function (*R* = −0.68) (Fig. 2D), with SI contributing to the largest proportion of variance explained (PVE = 0.35, *P* < 2e−16, Supplementary Table 3). It also indicated that global cognitive function in this cohort was positively correlated with education (PVE = 0.0020, *P* = 5.57e−3), inversely correlated with APOE4 allele count (PVE = 0.035, *P* = 5.00e−6), a diagnosis of Parkinson’s disease (PVE = 0.042, *P* = 7.49e−7), neocortical Lewy body disease (PVE = 0.016, *P* = 5.49e−4), hippocampal sclerosis (PVE = 0.014, *P* = 1.03e−3) and marginally age (PVE = 0.0047, *P* = 9.89e−2). When the two termini which were used in the training process were excluded from the linear regression model, we still observed strong correlations between SI and all neuropathology biomarkers and clinical severity (*P* < 0.1), especially for global cognitive function (PVE = 0.15, *P* = 3.5e−7 for SI, *R* = −0.55 for the model, Supplementary Tables 3 and 4 and Fig. 4), demonstrating the generality of the model outside the training data.

### Model achieved comparably strong performance in MAYO/MSBB cohorts

**Figure 3:**
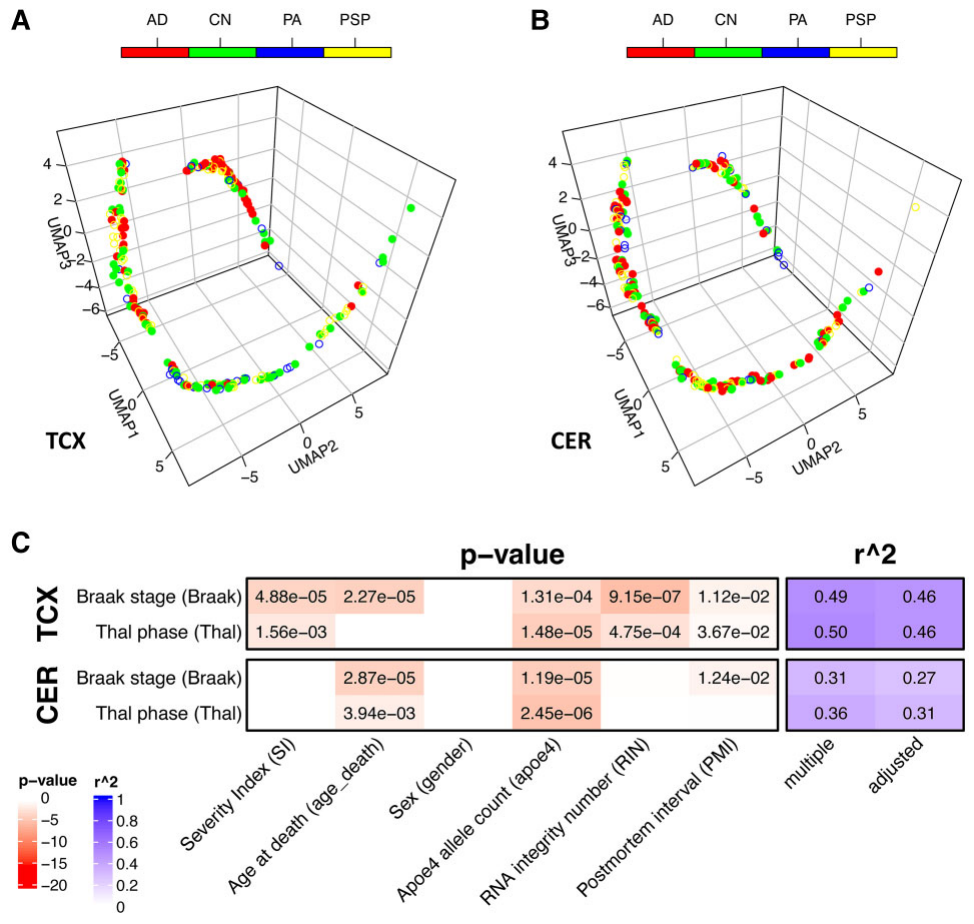
**The pseudo-temporal trajectory for the transcriptomes from two brain regions of MAYO cohort and the SI correlation with phenotypic data generated by applying the trained deep learning model and mapping to the same 3D space as ROSMAP**. AD, Alzheimer’s disease; CN, control; PA, pathological ageing; PSP, progressive supranuclear palsy. (**A**) The trajectory for transcriptome from TCX. (**B**) The trajectory for transcriptome from CER. (**C**) The model metrics for the linear regression between SI and the neuropathological biomarkers by different brain regions, with *P*-values shown in cells with *P* < 0.05. Detailed model metrics are reported in Supplementary Table 5.

**Figure 4:**
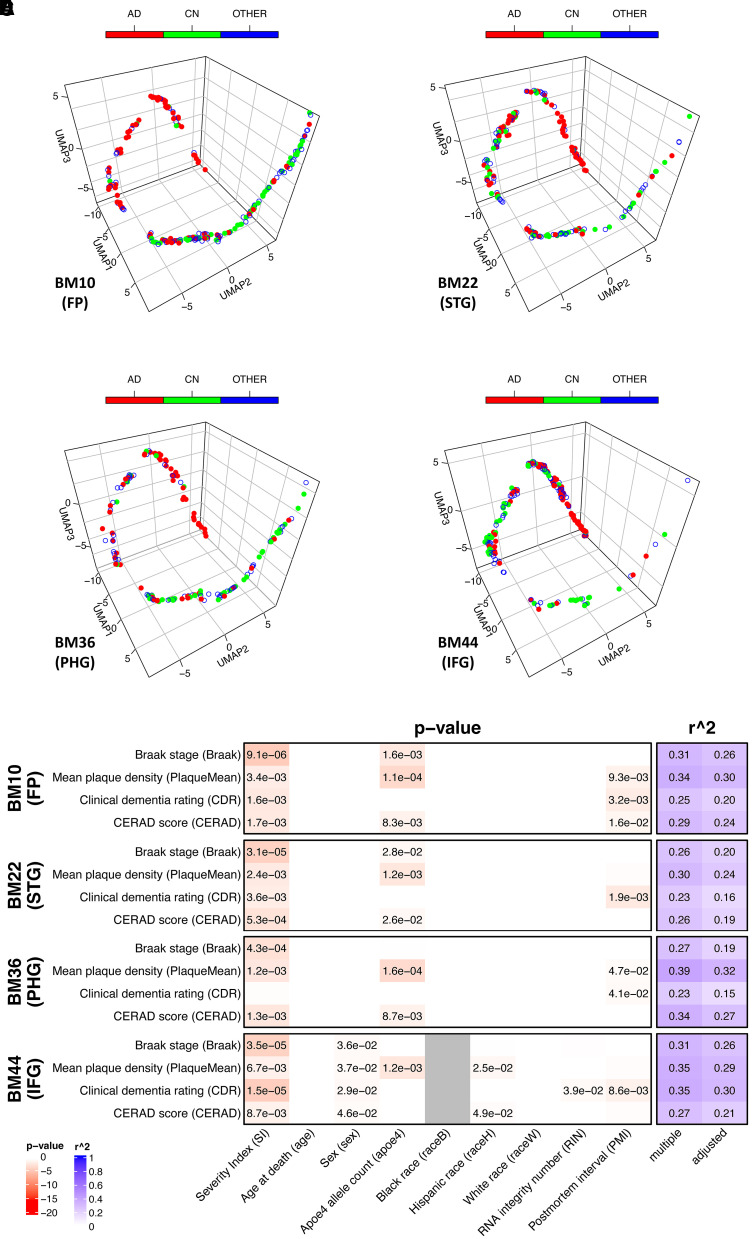
**The pseudo-temporal trajectory from the transcriptomes of four brain regions of MSBB cohort and the SI correlation with phenotypic data by applying the trained deep learning model and mapping to the same 3D space as ROSMAP data**. AD, Alzheimer’s disease (CDR ≥ 1 and Braak ≥ 4 and CERAD ≤ 2); CN, control (CDR ≤ 0.5 and Braak ≤ 3 and CERAD ≥ 3); OTHER, all other subjects. (**A**–**D**) The trajectory for transcriptomes from regions BM10/20/36/44, respectively. (**E**) The model metrics for the linear regression between SI and all the neuropathological and clinical biomarkers, by different brain regions with *P*-values shown in cells with *P* < 0.05. Grey cells indicate no data. Detailed model metrics are reported in Supplementary Table 6.

The model was applied to the harmonized transcriptomic data from both the MAYO and MSBB cohorts. Data from the MAYO cohort came from two different brain regions: TCX and CER. After projecting into the same 3D UMAP space, the subject distributions along the trajectories in the two different brain regions showed different patterns (Fig. 3A and B). For TCX, it showed the distributions of different locations for Alzheimer’s disease versus control subjects along the trajectory similar to those from ROSMAP data, while this was not observed for CER as one would expect. It was also confirmed by the results obtained from linear regression of the SI versus pathological biomarkers (Braak and Thal scores, Fig. 3C). Only in the TCX samples were the SIs found to be significantly correlated with both Braak (*P* = 4.88e−5) and Thal scores (*P* = 1.56e−3). Again the model explained a large amount of variance overall for both biomarkers, with *R* = 0.68. For the MSBB cohort, the same model was applied to the gene expression profile of all four sampled regions [FP (BM10), STG (BM22), PHG (BM36) and IFG (BM44)] and all regions show similar albeit slightly different trajectories, with the SI consistently significantly correlated with all the neuropathological and clinical biomarkers (Braak score, PlaqueMean, CDR scale and CERAD score, Fig. 4).

### Network analysis identified four major gene modules for disease progression

**Figure 5:**
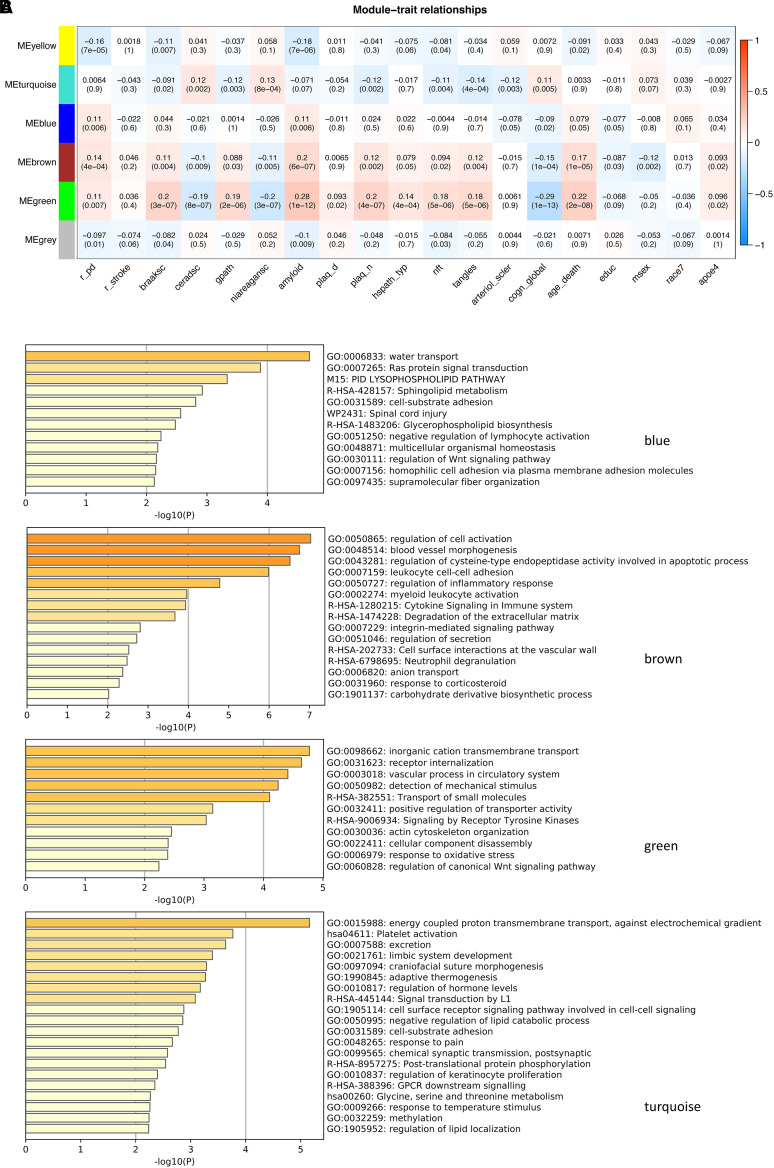
**Network and gene set functional enrichment analysis of the index genes**. (**A**) Module trait relationship between the eigengenes of the six co-expression modules and individual traits. (**B**) Functional enrichments for the index genes present in the four modules.

A clear bimodal distribution on the logarithm scale was observed in the weight distribution for the 15 582 genes in the deep learning model (Supplementary Fig. 2A). The cut-off was set at 1.6e−4, which generated 593 genes as the significant genes (index genes) associated with Alzheimer’s disease progression (Supplementary Table 7). The distribution of these genes showed some, though not complete overlap with those differentially expressed genes (DEGs) identified in previous work for ROSMAP cohort alone (syn8456629, Supplementary Fig. 2B), or from the AMP-AD meta-analysis (syn11914606, Supplementary Fig. 2C), as unlike DEGs, some of these index genes may have smaller fold change (log_2_FC), or not pass the significant *P*-value cut-off in comparison between Alzheimer’s disease versus control. Based on network analysis for these 593 genes’ expression profiles, six co-expression modules were identified, with four of them showing significant correlation with multiple phenotypes (Fig. 5A). Among them, the green module (*n* = 41) is significantly correlated with all the neuropathological and clinical biomarkers, while the turquoise module (*n* = 308) was found to be especially significantly correlated with tangles and the brown module (*n* = 61) with amyloid. Notably, the turquoise module’s directions of correlations with the pathological traits were reversed with the other three, although all showed significant contributions to cognitive functions. The yellow module (*n* = 53) was only significantly correlated with the diagnosis of Parkinson’s disease and amyloid, while the grey module (*n* = 63) showed little correlation with any of the pathological phenotypes as expected. We also decomposed the contributions of each module to the SI and found that the SIs derived for turquoise and green modules showed the strongest concordance with all the biomarkers and cognitive function scores (Supplementary Fig. 3 and Table 8).

**Figure 6:**
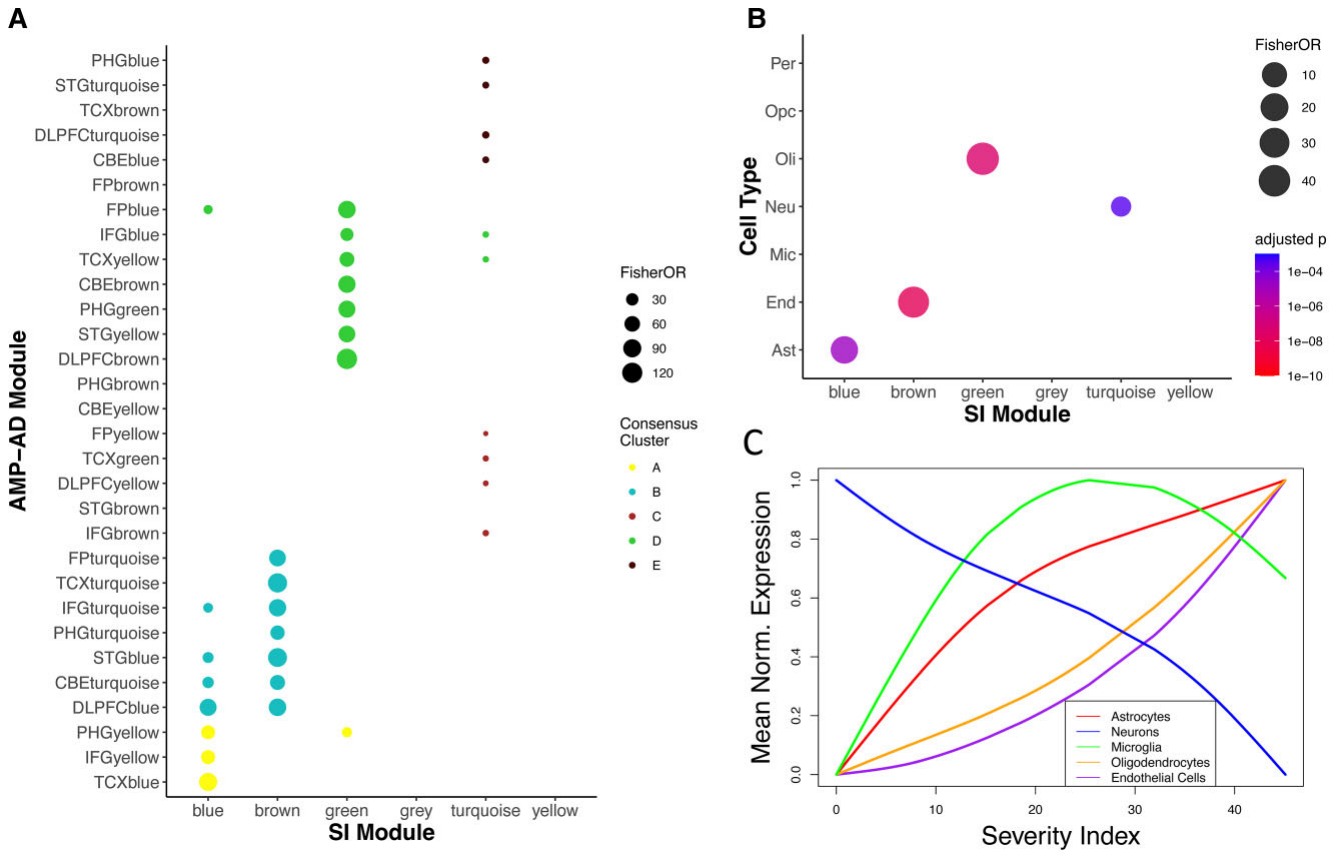
**Gene set overlaps with known gene sets for the index genes**. (**A**) The modules were examined for overlap (Fisher’s exact test) with the gene sets from the mega analysis of the AMP-AD consensus RNA-seq co-expression modules.^23^ Overlaps were shown for those adjusted *P* (Bonferroni correction) < 0.001. (**B**) Cell-type enrichments using Fisher’s exact test of gene set overlap of the modules with cell-type-specific gene sets from human reference single-cell RNA-seq data.^37,38^ Overlaps were shown for those adjusted *P* (Bonferroni correction) < 0.001. Ast, astrocytes; End, endothelial cells; Mic, microglia; Neu, neurons; Oli, oligodendrocytes; Opc, oligodendrocyte progenitor cells; Per, pericytes. (**C**) Cell-type marker gene expression signatures as a function of SI. The mean expression of cell markers for astrocytes, neurons, microglia, oligodendrocytes and endothelial cells were plotted and coloured, respectively.

Functional enrichment of the genes present in the four key modules showed that they were implicated in different processes. For the turquoise module, the enriched terms were related to metabolism and hormone activities; for the brown module, they were related to vascular dysfunction (Fig. 5B). Green and blue modules were implicated in metabolic abnormalities. Interestingly, the four modules were found to overlap mostly with the consensus cluster A (for blue), cluster B (for brown), cluster D (for green) and clusters C and E (for turquoise), respectively, from the meta-analysis of the Alzheimer’s disease human brain transcriptome^23^ (Fig. 6A) and concordantly, the three upregulated modules were each enriched for astrocytes (blue), oligodendrocytes (green) and endothelial cells (brown), and the downregulated turquoise module was enriched in neurons (Fig. 6B). Their overlaps with the curated Alzheimer’s disease gene sets from known databases and the gene sets from an individual cohort study in the AMP-AD consortium confirmed this observation (Supplementary Fig. 5).

The change of cell-type markers as a function of SI along disease progression (Fig. 6C) recapitulates known cellular changes such as neurodegeneration and gliosis. In addition, it suggests that microgliosis and astrogliosis happen earlier in the disease, while oligodendrocytes and endothelial cells are activated at a faster pace at the late stage. Interestingly, marker genes in microglia do not express monotonically in the process, which is especially prominent in males (Supplementary Fig. 7).

## Discussion

It is increasingly accepted that Alzheimer’s disease and Alzheimer’s disease-related dementias (AD/ADRD) are a spectrum of related diseases that have similar clinical and neuropathological manifestations.^40^ The progressive nature and relatively long deteriorating course warrant the study of the disease as a continuum instead of discrete states. However, the lack of longitudinal data from brain tissues of the same individuals prompts recent studies to model the gene expression dynamics of Alzheimer’s disease as pseudo-temporal trajectories, using data collected from post-mortem brain tissues in large cohorts, with various degrees of neurodegeneration and dementia severity. These unsupervised machine learning approaches provide novel insights into the progressive nature of Alzheimer’s disease and demonstrate that population-level cross-sectional transcriptomic data could be capitalized to capture the evolution of multiple Alzheimer’s disease-related neuropathology or cognitive impairment. In this work, we present a deep learning framework, which includes supervised classification and unsupervised dimension reduction of transcriptomic data to derive a trajectory that strongly mirrors Alzheimer’s disease-specific severity, since we have used the neuropathologically confirmed Alzheimer’s disease and control as the two termini to train the model. The SI defined by the distance along a trajectory could be utilized as a metric to evaluate the progression and staging of Alzheimer’s disease. The transcriptomic signature identified by the model sheds new light on the evolution of the gene expression profile across the disease course and illustrates the utility of deep learning approaches for the investigation of neurodegenerative diseases such as Alzheimer’s disease.

Notably, the deep learning component in our framework consists of a neural network of only three layers. There are two reasons we chose such a network. Firstly, the learning data sets from ROSMAP are composed of only 243 neuropathologically confirmed Alzheimer’s disease and control subjects. Although this is one of the largest post-mortem brain transcriptomic data sets of LOAD cohorts publicly available to date, more learning layers on this scale of data do not necessarily produce better results, as four or more layers in the current model did not improve the model’s performance by our tests. Conversely, a single-hidden layer in the model enables a straightforward interpretation of the embedding of the input gene features, which facilities the identification of index genes for downstream analysis. With the rapidly accelerating generation of multi-omic data for Alzheimer’s disease, this current framework could be easily extended to include additional layers for exploiting larger data sets, with both more samples or genomic features, and other phenotypic data types.

The original trajectory derived for the DLPFC tissues from the ROSMAP cohort suggests that the course of Alzheimer’s disease may be characterized by the multidimensional, non-linear nature of transcriptome dynamics in the neurodegenerative process. The neuropathologically confirmed Alzheimer’s disease and control subjects were mostly clustered in two corners, with relatively few subjects located between the two clusters along the trajectory. When other samples with various neuropathology and/or clinical diagnosis were mapped to the same space, they were distributed widely along the trajectory, with a considerable portion in-between the two clusters. SI still significantly predicted cognitive function (*P* = 3.5e−7) and neuropathology (*P* < 7e−2) for the other samples after excluding the Alzheimer’s disease/control termini used in the training process, demonstrating the validity of the model for the general dementia population. This was further demonstrated by the application of the model to the external data sets in the AMP-AD consortium, the MAYO and MSBB RNA-seq profiles. For all the brain regions known to be susceptible to Alzheimer’s disease-related pathology (TCX and FP/PHG/IFG/STG), the trajectories showed differentiating clusters of Alzheimer’s disease and control subjects. In stark contrast, for the transcriptome in CER, which possesses a distinct cellular architecture and is comparatively spared by Alzheimer’s disease neuropathology, the subjects’ locations are randomly distributed along the trajectory. Quantitatively, SIs derived from the trajectories confirmed the observation, with all of them correlating with the biomarkers closely except those for CER. Notably, thanks to the detailed neuropathological and clinical characterizations in the ROSMAP cohort enabling a covariate analysis in the linear regression model of SI against global cognitive function (Fig. 2C and Supplementary Table 3), cognitive impairment in the general dementia population is also partially attributable to multiple other comorbidities such as Parkinson’s and neocortical Lewy body disease, as well as hippocampal sclerosis and arteriolosclerosis. This underscores the heterogeneity of the broad dementia spectrum and the urgent need to dissect the spectrum by distinguishing the Alzheimer’s disease-specific pathology and those caused by other related diseases for precision dementia diagnosis and treatment.

Deep learning methods have been demonstrated recently to be able to capture complex, non-linear transcriptomic features that are not learned using conventional gene expression data analysis methods in Alzheimer’s disease cohorts.^41^ In our model, it searches the data for correlated features and combines them by amplifying the underlying signals with adjustable weights and the sigmoid function, so it can extract the genomic features most pertinent to the questions we are asking, that is, the coordinated transcriptomic signature differentiating definitive Alzheimer’s disease and control. The model is completely portable and applicable to any gene expression data, and the reproducible significant results in external data sets from pathologically affected tissues manifest that the index genes indeed play significant roles in the progression of LOAD.

The index genes identified by the deep learning model have unique implications, in our understanding of Alzheimer’s disease aetiology, as well as the pursuit of novel therapeutics. They have some overlap, but considerably differ from those DEGs identified by DE analysis. Recently, it has been demonstrated that certain gene expression profiles from RNA-seq experiments are generic, with a high probability of DE across a wide variety of biological conditions, so their specificity related to disease mechanisms has been challenged.^42^ Additionally, DEGs are those passing certain statistical cut-offs systematically for both fold change and *P*-values selected as the ‘hit list’ for further interpretation and validation. The cut-offs are arbitrarily set following a convention [e.g. fdr < 0.05, |log_2_FC| > 0.263 in the DEG analysis for the ROSMAP data set (syn8456629)], so it might not be able to capture subtle, intrinsic and coordinated gene expression signatures due to disease pathology in the high-dimensional data, especially from bulk tissues.^41,43^ This is further demonstrated by the fact that neither DEGs identified by ROSMAP data set alone (syn8456629) nor those from the AMP-Alzheimer’s disease meta-analysis (syn11914606) could fully reproduce the progressive trajectory in any of the three transcriptomic data sets, especially the external data sets when applied with the model (results not shown).

Among the six co-expression modules from the index genes, blue, brown, green and turquoise modules are significantly correlated with Alzheimer’s disease phenotypical hallmarks, with the first three upregulated in the progression, while turquoise genes downregulated. Together with the cell-type enrichment analysis showing the three modules are enriched in astrocytes, oligodendrocytes and endothelial cells, respectively, and turquoise module enriched in neurons, this is consistent with the results obtained by the recent work of neurodegeneration pseudotime estimation^5^ and cellular composition deconvolution,^43^ which shows a reduction in the neuronal populations as Alzheimer’s disease progresses, and an increase in expression associated with activation of endothelial and glial cells, as also demonstrated by the change of mean expression for the marker genes of each cell type along Alzheimer’s disease progression. Interestingly, the transcriptomic signatures obtained from our study show high similarity with the signatures obtained from a recent single-nucleus transcriptome analysis from the prefrontal cortical samples of Alzheimer’s disease patients and normal control subjects,^38^ where higher proportion of endothelial nuclei was sampled and dysregulated pathways are associated with blood vessel morphogenesis, angiogenesis and antigen presentation. Notably, these functions are also implicated in the top common blood–brain functional pathways relevant for LOAD progression in the recent study of gene expression trajectories in Alzheimer’s disease.^5^ In addition, the enriched functions of proton transport implicated in mitochondrial functions and cell signalling pathway in the turquoise module were also found to be associated with the overlapped DEGs in neurons from two independent single nuclei transcriptomic studies from Alzheimer’s disease patients.^38,44^ Strikingly, although the brown module is mostly overlapped with the consensus module cluster B from the AMP-AD transcriptome meta-analysis^23^ (Fig. 6A), it is not enriched in microglia but endothelial cells (Fig. 6B). It is most likely a submodule in the cluster, which represents the signatures from endothelial cells. Likewise, the turquoise module as a subset of the consensus module cluster E shows comparatively poor enrichment for cell-type expression signatures and were not well annotated or represented among curated Alzheimer’s disease pathways (Supplementary Fig. 5). These results collectively suggest that the transcriptomic signatures identified by the deep learning framework constitute intrinsic molecular changes at the cellular level associated with Alzheimer’s disease’s progression. Whether the changes are the drivers of the progression or just physiological responses accompanying the progression awaits further examination. With the identification of the four modules each enriched in a specific cell type and strongly associated with Alzheimer’s disease severity, the future work will be a scrutiny of these genes for their roles in Alzheimer’s disease’s progression.

It is more and more evident that sexual dimorphism plays an important role in Alzheimer’s disease’s development and progression.^45^ In the recent work by Mukherjee *et al.*^5^ where unsupervised learning methods were applied to the ROSMAP transcriptome data, predictive pseudotimes were only observed for female samples, highlighting great diversity of the gene expression profiles between the sexes. While we did not explicitly include sex as a feature node in the deep learning model, our analysis showed that the sex effect has been modelled through the expression levels of sex markers such as XIST.^46^ When we compare SI with the pseudotime obtained by unsupervised learning on the same subjects as reported in the work of Mukherjee *et al.*, higher degree of concordance is obtained for the female samples than the male (Supplementary Fig. 6A). SI was found to be significantly associated with the neuropathological and clinical biomarkers in both sexes, by both linear and logistic regressions (Supplementary Fig. 6B and C). In the plots stratified by sex of the mean marker gene expression as a function of SI, we observe not only consistent overall patterns in both sexes, but also distinct curves for some cell types, for example, neuron and microglia (Supplementary Fig. 7B and C). Neuronal degeneration occurs earlier and faster in female, although eventually, both sexes converge at a similar total loss. For microglia, the change is not monotonic, especially in male. These observations highlight important cell-type-specific contributions to Alzheimer’s disease progression in different sexes, which has drawn considerable research efforts in recent years.^47,48^ Lastly, sex has also been considered as a covariate in the linear regression of SI against all the neuropathological and clinical biomarkers in all three cohorts. They are all not significant except in the IFG region from the MSBB cohort (Fig. 4E and Supplementary Table 6), where female sex is associated with higher clinical and pathological severity. All of the above work collectively demonstrates that our model captures generalized transcriptomic features present in both sexes for most Alzheimer’s disease-affected brain regions like DLPFC, while in specific brain region like IFG, there are additional sex effects that await further investigation.

From the trajectories derived for different Alzheimer’s disease-affected brain regions and their model metrics (e.g. MSBB cohort, Fig. 4 and Supplementary Table 6), it is evident that there are perceptible although subtle variations within their expression profiles, consistent with the observations from multiple analyses of transcriptomic data for the cohort.^49,50^ This highlights the necessity of analysing tissue and region-specific data sets to better understand interactions between a brain region and molecular disease states within Alzheimer’s disease. For instance, with preliminary validation in this work, a similar deep learning framework could be designed specifically to dissect transcriptomes from peripheral tissues such as blood of Alzheimer’s disease cohorts to seek highly sensitive and specific targets, to complement any biomarkers currently being actively pursued for early diagnosis of the devastating disease. Similarly, the application of such an approach has broad utility for use with any high-dimensional multi-omics data such as proteomics, metabolomics and epigenomics, thus opening another channel for the application of artificial intelligence in the genomics field for pursuing early diagnosis and effective treatment of neurodegenerative diseases.

## Supplementary Material

fcab293_Supplementary_Data

## Abbreviations

AD/ADRD =: Alzheimer’s disease and Alzheimer's disease-related dementias
AMP-AD =: Accelerating Medicines Project for Alzheimer’s Disease
BM =: Brodmann area
CDR =: clinical dementia rating
CER =: cerebellum
CERAD =: Consortium to Establish a Registry for Alzheimer’s Disease
CPM =: counts per million reads
DEG =: differentially expressed gene
DLPFC =: dorsolateral prefrontal cortex
FP =: frontal pole
IFG =: inferior frontal gyrus
LOAD =: late-onset Alzheimer’s disease
MCI =: mild cognitive impairment
MSBB =: Mount Sinai Brain Bank
NIA =: National Institute on Aging
PCA =: principal components analysis
PHG =: parahippocampal gyrus
PMI =: post-mortem interval
PVE =: proportion of variance explained
RADC =: Rush Alzheimer’s Disease Center
RIN =: RNA integrity number
ROSMAP =: Religious Orders Study and Memory and Aging Project
SD =: standard deviation
SI =: severity index
STG =: superior temporal gyrus
TCX =: temporal cortex
UMAP =: Uniform Manifold Approximation and Projection.
