# Supplementary material for: Deep learning-based brain transcriptomic signatures associated with the neuropathological and clinical severity of Alzheimer’s disease
Source: Brain Commun. 2021 Dec 14;4(1):fcab293. doi: 10.1093/braincomms/fcab293 (PMC8728025; doi:10.1093/braincomms/fcab293)
Supplement: fcab293_Supplementary_Data [file fcab293_supplementary_data.zip › SupplementalTables.pdf]

Supplemental Table 1 Demographic information for all the subjects included in RNA-seq data used in this study.

Study	Sample size#	Tissue	AD		control		other**	
			gender (M/F)	age* (mean/SD)	gender (M/F)	age* (mean/SD)	gender (M/F)	age* (mean/SD)
ROSMAP	634	DLPFC	46/110	91.0/5.7	39/48	84.1/6.8	143/248	88.8/6.5
MAYO	263	CER	32/47	82.5/7.7	38/36	82.4/8.3	61/49	76.7/7.6
	266	TCX	31/49	82.6/7.7	38/36	82.7/8.4	62/50	77.0/7.8
MSBB	214	FP (BM10)	27/58	84.4/6.6	25/29	80.6/8.6	21/54	85.5/6.0
	191	STG (BM22)	29/50	83.0/7.2	19/26	80.1/9.4	17/50	85.6/6.2
	162	PHG (BM36)	18/44	84.4/6.9	23/21	79.5/9.5	17/39	84.6/6.7
	187	IFG (BM44)	24/49	84.0/6.9	23/21	80.1/9.2	18/52	85.6/6.1

* All those with age > 90 in MAYO/MSBB cohort were calculated as 90
** All diagnosis confirmed by neuropathological assessments
# Sample size after outlier removal in RNA-seq normalization

Supplemental Table 2 Description of the variables used in the linear regression model for ROSMAP cohort.

category		code	description	value range	note
diagnosis		cogdx	Final consensus cognitive diagnosis	1,2,3,4,5,6	Clinical consensus diagnosis of cognitive status at time of death
variables used in linear regression model	dependent variable, AD-related quantitative measurements	braaksc	Braak stage	0,1,2,3,4,5,6	Semiquantitative measure of neurofibrillary tangles
		ceradsc	CERAD score	1,2,3,4	Semiquantitative measure of neuritic plaques
		niareagansc	NIA-Reagan diagnosis of AD	1,2,3,4	Consensus postmortem diagnosis based on both neurofibrillary tangles (Braak) and neuritic plaques (CERAD)
		gpath	Global AD pathology burden	0.000 ~ 3.200	Quantitative summary of three AD pathologies: neuritic plaques (n), diffuse plaques (d), and neurofibrillary tangles (nft)
		amyloid	Overall amyloid level	0.000 ~ 19.925	Overall amyloid level - Mean of 8 brain regions
		plaq_d	Diffuse plaque burden	0.000 ~ 4.912	Diffuse plaque summary based on 5 regions
		plaq_n	Neuritic plaque burden	0.000 ~ 5.004	Neuritic plaque summary based on 5 regions
		nft	Neurofibrillary tangle burden	0.000 ~ 6.099	Neurofibrillary tangle summary based on 5 regions
		tangles	Tangle density	0.000 ~ 78.523	Tangle density - Mean of 8 brain regions
		cogn_global	Global cognitive function	-4.239 ~ 1.224	Z score from 19 cognitive tests
	independent variable, non-AD quantitative measurements	SI	Severity Index	0.00 ~ 45.10	defined in this work
		age_death	Age at death	67.37 ~ 108.28	
		educ	Year of education	3 ~ 28	
		msex	Sex	0,1	
		race7	Racial group	1,2,3,4,5,6,7	categorical
		apoe4	Apoe4 allele count	0,1,2	
		RIN	RNA integrity number	5.0 ~ 9.9	
		PMI	Postmortem interval	1.0 ~ 40.8	
		r_pd	Clinical diagnosis of Parkinson's Disease	1,2,3,4	
		r_stroke	Clinical stroke diagnosis	1,2,3,4	
		dlbdx	Lewy body disease	0,1,2,3	categorical; 4 stages of pathologic diagnosis of Lewy body diseases
		hspath_typ	Hippocampal sclerosis	0,1	Definite presence of typical hippocampal sclerosis
		arteriol_scler	Arteriolosclerosis	0,1,2,3	4 stages of arteriolosclerosis

Supplemental Table 3 Model metrics for the linear regression between global cognitive function and the dependent variables (as defined in Supplemental Table 2) stratified by diagnosis groups in ROSMAP DLPFC samples. Cells with significant p values (< 0.05) are shown in bold.

target = global cognitive function	all subjects (n = 634)						all OTHER subjects (n = 391)						all AD and CN (n = 243)					
	Estimate	Std. Error	t value	Pr(>|t|)	PVE	sig. code	Estimate	Std. Error	t value	Pr(>|t|)	PVE	sig. code	Estimate	Std. Error	t value	Pr(>|t|)	PVE	sig. code
(Intercept)	-0.347	0.779	-0.445	6.56E-01			-1.997	0.937	-2.131	**3.41E-02**		*	1.683	1.349	1.247	2.14E-01		
SI	-0.036	0.003	-11.345	**< 2E-16**	34.96	***	-0.021	0.004	-5.237	**3.50E-07**	15.23	***	-0.050	0.005	-9.540	**< 2E-16**	57.45	***
age_death	-0.011	0.007	-1.654	9.89E-02	0.47	.	-0.002	0.008	-0.266	7.91E-01	0.17		-0.028	0.012	-2.374	**1.89E-02**	1.60	*
educ	0.034	0.012	2.787	**5.57E-03**	0.20	**	0.024	0.015	1.645	1.01E-01	0.07		0.056	0.022	2.575	**1.11E-02**	1.43	*
msex	-0.099	0.082	-1.209	2.28E-01	0.38		-0.059	0.097	-0.610	5.43E-01	0.37		-0.050	0.141	-0.353	7.25E-01	0.05	
race7	-0.018	0.303	-0.060	9.52E-01	0.08		-0.278	0.330	-0.841	4.01E-01	0.35		0.072	0.602	0.119	9.06E-01	0.02	
apoe4	-0.387	0.084	-4.627	**5.00E-06**	3.48	***	-0.262	0.102	-2.575	**1.06E-02**	2.82	*	-0.323	0.139	-2.314	**2.21E-02**	1.33	*
RIN	-0.041	0.042	-0.984	3.25E-01	0.37		0.042	0.049	0.853	3.95E-01	0.04		-0.108	0.075	-1.442	1.52E-01	1.05	
PMI	0.007	0.009	0.835	4.04E-01	0.00		0.014	0.011	1.323	1.87E-01	0.08		0.004	0.015	0.256	7.99E-01	0.01	
r_pd	0.262	0.052	5.027	**7.49E-07**	4.21	***	0.281	0.058	4.804	**2.70E-06**	8.17	***	0.163	0.096	1.690	9.33E-02	0.77	.
r_stroke	0.090	0.059	1.539	1.25E-01	0.19		0.111	0.062	1.791	7.46E-02	0.94	.	0.078	0.126	0.618	5.38E-01	0.01	
dlbdx3	-0.464	0.133	-3.484	**5.49E-04**	1.60	***	-0.372	0.169	-2.193	**2.92E-02**	1.43	*	-0.515	0.202	-2.552	**1.18E-02**	1.72	*
hspath_typ	-0.555	0.168	-3.307	**1.03E-03**	1.43	**	-0.873	0.228	-3.827	**1.64E-04**	4.15	***	-0.180	0.245	-0.738	4.62E-01	0.12	
arteriol_scler	-0.054	0.038	-1.406	1.60E-01	0.26		-0.039	0.046	-0.847	3.98E-01	0.19		-0.038	0.063	-0.597	5.52E-01	0.09	
Multiple R^2	0.476						0.340						0.656					
Adjusted R^2	0.457						0.300						0.620					

Signif. codes: 0 ‘***’ 0.001 ‘**’ 0.01 ‘*’ 0.05 ‘.’ 0.1 ‘ ’ 1

Supplemental Table 4 Model metrics (p values and correlation coefficients) for the linear regression between all the neuropathological biomarkers and the dependent variables (as defined in Supplemental Table 2) stratified by diagnosis groups in ROSMAP DLPFC samples. Cells with significant p values (< 0.05) are shown in bold.

target	braaksc			ceradsc			niareagansc			gpath			amyloid		
p value	all	OTHER	AD/ CN	all	OTHER	AD/ CN	all	OTHER	AD/ CN	all	OTHER	AD/ CN	all	OTHER	AD/ CN
(Intercept)	1.40E-01	9.55E-01	1.56E-01	**5.53E-10**	**6.98E-05**	**5.06E-05**	**2.46E-12**	**1.87E-07**	**7.28E-05**	2.56E-01	6.72E-01	8.26E-01	4.92E-01	8.94E-01	9.73E-01
SI	**1.00E-14**	7.29E-02	**6.33E-16**	**2.04E-14**	**4.25E-03**	**1.21E-15**	**2.00E-16**	**1.13E-02**	**2.00E-16**	**2.00E-16**	**2.25E-03**	**7.31E-16**	**7.16E-12**	**2.54E-02**	**1.32E-12**
age_death	**2.71E-08**	**1.39E-04**	**1.22E-05**	**1.50E-02**	2.93E-01	**1.73E-02**	**7.65E-03**	**2.89E-02**	1.80E-01	2.17E-01	5.58E-01	3.55E-01	**4.39E-02**	4.45E-01	**3.61E-02**
educ	8.39E-02	9.18E-02	1.57E-01	2.45E-01	1.81E-01	5.31E-01	7.31E-01	3.31E-01	3.05E-01	8.36E-01	9.74E-01	6.14E-01	**1.90E-02**	1.43E-01	**1.80E-02**
msex	1.79E-01	1.63E-01	3.15E-01	5.04E-01	9.98E-01	3.65E-01	9.30E-01	6.51E-01	8.06E-01	7.30E-01	4.42E-01	9.18E-01	7.01E-01	9.37E-01	8.84E-01
race7	7.31E-01	6.63E-01	4.88E-01	4.24E-01	8.64E-01	4.63E-01	5.10E-01	5.91E-01	6.48E-01	8.93E-01	5.29E-01	7.63E-01	3.03E-01	6.52E-01	6.97E-01
apoe4	**5.66E-05**	**2.82E-02**	**2.57E-02**	**2.28E-07**	**1.45E-04**	**1.59E-03**	**3.26E-07**	**3.56E-04**	**2.88E-02**	**3.71E-12**	**1.84E-06**	**1.62E-05**	**8.60E-08**	**2.69E-04**	**3.97E-04**
RIN	3.20E-01	1.00E+00	2.73E-01	9.08E-02	5.49E-01	1.05E-01	3.08E-01	8.45E-01	4.47E-01	**6.14E-02**	4.90E-01	8.61E-02	9.92E-01	7.24E-01	8.13E-01
PMI	1.21E-01	**5.99E-02**	7.82E-01	**4.96E-02**	1.28E-01	3.42E-01	**4.84E-02**	8.32E-02	3.72E-01	**3.63E-02**	**1.62E-02**	8.77E-01	3.81E-01	1.14E-01	2.08E-01
r_pd	6.10E-01	1.48E-01	4.79E-01	8.19E-01	**6.85E-02**	**7.76E-03**	9.65E-01	4.20E-01	4.90E-01	8.99E-01	1.09E-01	9.52E-02	1.72E-01	**1.50E-02**	2.64E-01
r_stroke	3.02E-01	3.19E-01	1.96E-01	6.28E-01	3.36E-01	2.24E-01	8.26E-01	8.89E-01	3.02E-01	8.13E-01	5.48E-01	2.59E-01	3.78E-01	2.21E-01	3.63E-01
dlbdx3	7.54E-01	2.11E-01	**2.37E-02**	6.43E-01	5.69E-01	2.82E-01	8.12E-01	9.61E-01	1.00E+00	6.58E-01	7.63E-01	9.23E-01	4.20E-01	3.37E-01	5.37E-01
hspath_typ	2.49E-01	**1.04E-02**	6.98E-01	3.86E-01	1.69E-01	9.74E-01	9.89E-01	8.64E-01	5.67E-01	9.97E-01	8.43E-01	8.90E-01	5.11E-01	9.37E-01	1.46E-01
arteriol_scler	9.66E-01	2.28E-01	7.55E-02	4.99E-01	**2.03E-02**	7.23E-02	3.13E-01	**2.22E-02**	3.66E-01	2.59E-01	5.37E-02	5.99E-01	5.35E-02	1.19E-01	1.41E-01
Multiple R^2	0.33	0.19	0.67	0.27	0.15	0.64	0.31	0.14	0.63	0.32	0.16	0.61	0.26	0.15	0.57
Adjusted R^2	0.30	0.14	0.63	0.24	0.09	0.60	0.28	0.08	0.59	0.30	0.11	0.57	0.23	0.10	0.53

target	plaq_d			plaq_n			nft			tangles			cogn_global		
p value	all	OTHER	AD/ CN	all	OTHER	AD/ CN	all	OTHER	AD/ CN	all	OTHER	AD/ CN	all	OTHER	AD/ CN
(Intercept)	6.49E-01	9.46E-01	8.10E-01	2.18E-01	4.76E-01	9.58E-01	1.99E-01	4.00E-01	5.22E-01	2.19E-01	4.73E-01	2.99E-01	6.56E-01	**3.41E-02**	2.14E-01
SI	**3.24E-06**	**4.61E-02**	**4.27E-05**	**2.00E-16**	**3.64E-03**	**3.78E-16**	**2.00E-16**	**1.26E-02**	**1.08E-12**	**2.00E-16**	**1.31E-02**	**9.39E-14**	**2.00E-16**	**3.50E-07**	**2.00E-16**
age_death	2.49E-01	9.01E-01	1.60E-01	6.31E-01	9.97E-01	7.30E-01	1.14E-01	**4.47E-02**	6.15E-01	**1.90E-03**	**6.60E-03**	**2.14E-02**	9.89E-02	7.91E-01	**1.89E-02**
educ	2.53E-01	6.18E-01	4.55E-01	9.43E-01	4.45E-01	2.46E-01	**2.56E-02**	7.10E-02	2.35E-01	1.47E-01	9.32E-01	**3.68E-02**	**5.57E-03**	1.01E-01	**1.11E-02**
msex	9.74E-01	5.09E-01	4.16E-01	7.61E-01	1.83E-01	6.13E-01	3.20E-01	5.51E-02	7.94E-01	**3.26E-02**	**7.63E-03**	5.88E-01	2.28E-01	5.43E-01	7.25E-01
race7	9.02E-01	3.62E-01	2.34E-01	3.70E-01	7.16E-01	9.57E-01	8.13E-01	5.14E-01	6.26E-01	4.82E-01	2.62E-01	6.46E-01	9.52E-01	4.01E-01	9.06E-01
apoe4	**6.30E-07**	**5.59E-04**	**4.60E-04**	**1.47E-09**	**1.61E-06**	**2.03E-03**	**4.88E-08**	**3.74E-03**	**8.14E-04**	**1.24E-04**	1.52E-01	**6.77E-03**	**5.00E-06**	**1.06E-02**	**2.21E-02**
RIN	3.17E-01	6.73E-01	4.28E-01	**7.75E-02**	6.54E-01	**6.04E-02**	1.10E-01	5.56E-01	2.27E-01	1.95E-01	7.76E-01	6.85E-02	3.25E-01	3.95E-01	1.52E-01
PMI	1.71E-01	1.60E-01	6.49E-01	**6.76E-03**	**2.89E-03**	9.67E-01	3.22E-01	1.62E-01	5.83E-01	3.14E-01	1.38E-01	5.66E-01	4.04E-01	1.87E-01	7.99E-01
r_pd	2.83E-01	8.43E-01	**3.82E-02**	4.76E-01	**7.77E-03**	**2.57E-02**	2.92E-01	1.53E-01	6.75E-01	7.79E-01	4.33E-01	3.60E-01	**7.49E-07**	**2.70E-06**	9.33E-02
r_stroke	5.23E-01	6.00E-01	**6.96E-03**	3.13E-01	2.45E-01	8.35E-01	5.63E-01	8.85E-01	7.61E-01	9.99E-01	5.20E-01	9.95E-01	1.25E-01	7.46E-02	5.38E-01
dlbdx3	7.33E-01	6.73E-01	7.62E-01	2.60E-01	8.34E-01	1.11E-01	7.50E-01	4.99E-01	3.53E-01	4.73E-01	9.60E-01	2.38E-01	**5.49E-04**	**2.92E-02**	**1.18E-02**
hspath_typ	8.19E-01	4.83E-01	3.99E-01	5.33E-01	1.23E-01	2.98E-01	6.49E-01	8.22E-02	9.13E-01	1.66E-01	**2.31E-02**	4.49E-01	**1.03E-03**	**1.64E-04**	4.62E-01
arteriol_scler	**5.68E-02**	**1.87E-02**	8.92E-01	4.92E-01	9.93E-02	4.61E-01	8.08E-01	8.31E-01	4.08E-01	9.43E-01	4.19E-01	6.78E-01	1.60E-01	3.98E-01	5.52E-01
Multiple R^2	0.16	0.10	0.42	0.29	0.20	0.57	0.30	0.15	0.52	0.30	0.16	0.56	0.48	0.34	0.66
Adjusted R^2	0.13	0.05	0.36	0.26	0.15	0.53	0.27	0.10	0.47	0.27	0.11	0.51	0.46	0.30	0.62

Supplemental Table 5 Model metrics (p values and correlation coefficients) for the linear regression between neuropathological biomarkers and the dependent variables for the two brain regions in Mayo samples. Cells with significant p values (< 0.05) are shown in bold.

brain region	TCX		CER	
target	Braak	Thal	Braak	Thal
(Intercept)	**8.24E-07**	**2.36E-03**	**1.96E-02**	2.04E-01
SI	**4.88E-05**	**1.56E-03**	6.47E-01	4.61E-01
age_death	**2.27E-05**	1.69E-01	**2.87E-05**	**3.94E-03**
gender	9.81E-01	8.18E-01	8.07E-01	8.60E-01
apoe4	**1.31E-04**	**1.48E-05**	**1.19E-05**	**2.45E-06**
RIN	**9.15E-07**	**4.75E-04**	8.05E-02	5.11E-01
PMI	**1.12E-02**	**3.67E-02**	**1.24E-02**	8.52E-02
Multiple R^2	0.49	0.50	0.31	0.36
Adjusted R^2	0.46	0.46	0.26	0.31

Supplemental Table 6 Model metrics (p values and correlation coefficients) for the linear regression between neuropathological and clinical biomarkers and the dependent variables for the four brain regions in MSBB samples. Cells with significant p values (< 0.05) are shown in bold.

brain region	BM10 (FP)				BM22 (STG)				BM36 (PHG)				BM44 (IFG)			
target	Braak	Plaque Mean	CDR	CERAD	Braak	Plaque Mean	CDR	CERAD	Braak	Plaque Mean	CDR	CERAD	Braak	Plaque Mean	CDR	CERAD
(Intercept)	9.24E-01	3.91E-01	7.52E-01	**2.61E-02**	8.78E-01	6.30E-01	1.06E-01	5.90E-02	9.43E-01	7.98E-01	3.79E-01	**3.35E-02**	1.65E-01	1.85E-01	**4.55E-02**	**4.75E-02**
SI	**9.11E-06**	**3.42E-03**	**1.61E-03**	**1.72E-03**	**3.09E-05**	**2.36E-03**	**3.60E-03**	**5.28E-04**	**4.29E-04**	**1.17E-03**	6.74E-02	**1.28E-03**	**3.51E-05**	**6.69E-03**	**1.52E-05**	**8.74E-03**
age	2.08E-01	9.61E-01	9.40E-01	3.68E-01	6.58E-01	8.36E-01	2.51E-01	8.80E-01	3.43E-01	5.37E-01	7.06E-01	3.16E-01	9.87E-01	8.38E-01	2.80E-01	6.86E-01
sex	3.39E-01	2.36E-01	2.21E-01	2.39E-01	1.53E-01	1.71E-01	3.98E-01	2.08E-01	2.62E-01	7.45E-01	3.57E-01	6.61E-01	**3.56E-02**	**3.68E-02**	**2.93E-02**	**4.57E-02**
apoe4	**1.65E-03**	**1.10E-04**	5.80E-01	**8.30E-03**	**2.81E-02**	**1.22E-03**	7.22E-01	**2.64E-02**	9.06E-02	**1.58E-04**	5.11E-01	**8.67E-03**	1.01E-01	**1.23E-03**	4.32E-01	1.71E-01
raceB	8.50E-01	5.86E-01	9.08E-01	8.29E-01	9.62E-01	4.48E-01	5.57E-01	5.89E-01	9.25E-01	9.02E-01	7.13E-01	9.20E-01	-	-	-	-
raceH	4.24E-01	6.92E-01	7.01E-01	5.13E-01	5.67E-01	9.55E-01	9.49E-01	7.32E-01	6.24E-01	6.20E-01	8.10E-01	5.73E-01	8.43E-02	**2.50E-02**	3.05E-01	**4.90E-02**
raceW	8.37E-01	4.79E-01	6.83E-01	7.79E-01	9.02E-01	4.33E-01	4.98E-01	5.88E-01	9.02E-01	7.56E-01	6.86E-01	8.38E-01	6.73E-01	3.55E-01	9.60E-02	5.96E-01
RIN	7.37E-01	3.80E-01	3.34E-01	7.02E-01	4.75E-01	4.88E-01	3.04E-01	8.85E-01	6.69E-01	5.61E-01	1.77E-01	7.72E-01	7.59E-02	2.12E-01	**3.88E-02**	3.41E-01
PMI	3.34E-01	**9.29E-03**	**3.25E-03**	**1.61E-02**	6.18E-01	5.88E-02	**1.93E-03**	1.14E-01	8.18E-01	**4.71E-02**	**4.13E-02**	1.02E-01	5.70E-01	6.06E-02	**8.55E-03**	6.04E-02
Multiple R^2	0.31	0.25	0.30	0.35	0.26	0.23	0.26	0.30	0.27	0.23	0.34	0.39	0.31	0.35	0.27	0.35
Adjusted R^2	0.26	0.20	0.24	0.30	0.20	0.16	0.19	0.24	0.19	0.15	0.27	0.32	0.26	0.30	0.21	0.30

Supplemental Table 7 Index genes and their weights contributing to the deep learning model.

ensembl ID	symbol	weight	module
ENSG00000088836	SLC4A11	1	green
ENSG00000131095	GFAP	0.8831	blue
ENSG00000274276	CBSL	0.76773	grey
ENSG00000196517	SLC6A9	0.75606	green
ENSG00000158169	FANCC	0.7318	green
ENSG00000168743	NPNT	0.66154	turquoise
ENSG00000125337	KIF25	0.63187	grey
ENSG00000262877	AC110285.2	0.6311	grey
ENSG00000134533	RERG	0.62733	blue
ENSG00000153822	KCNJ16	0.57374	blue
ENSG00000183090	FREM3	0.55655	turquoise
ENSG00000140368	PSTPIP1	0.52183	blue
ENSG00000182648	LINC01006	0.50898	turquoise
ENSG00000196415	PRTN3	0.50515	turquoise
ENSG00000115602	IL1RL1	0.43866	brown
ENSG00000205611	LINC01597	0.43353	turquoise
ENSG00000140678	ITGAX	0.40071	brown
ENSG00000189343	RPS2P46	0.39502	turquoise
ENSG00000225302		0.38756	grey
ENSG00000275830	AL355974.2	0.38418	brown
ENSG00000169031	COL4A3	0.36828	brown
ENSG00000166012	TAF1D	0.36241	turquoise
ENSG00000020577	SAMD4A	0.36187	brown
ENSG00000154864	PIEZO2	0.35731	green
ENSG00000105643	ARRDC2	0.34802	brown
ENSG00000206195	DUXAP8	0.33919	grey
ENSG00000111788	AC009533.1	0.33254	grey
ENSG00000136297	MMD2	0.30094	blue
ENSG00000253159	PCDHGA12	0.29011	blue
ENSG00000163319	MRPS18C	0.28927	turquoise
ENSG00000138778	CENPE	0.2886	yellow
ENSG00000139410	SDSL	0.28818	turquoise
ENSG00000148655	LRMDA	0.28607	grey
ENSG00000228314	CYP4F29P	0.28601	grey
ENSG00000136449	MYCBPAP	0.28394	turquoise
ENSG00000198830	HMGN2	0.28372	turquoise
ENSG00000142910	TINAGL1	0.27878	brown
ENSG00000146250	PRSS35	0.27569	blue
ENSG00000157005	SST	0.26298	turquoise
ENSG00000151233	GXYLT1	0.25542	blue
ENSG00000125534	PPDPF	0.24935	turquoise
ENSG00000136732	GYPC	0.24319	brown
ENSG00000176387	HSD11B2	0.24242	grey
ENSG00000143546	S100A8	0.24016	brown
ENSG00000246379	AC007495.1	0.23992	yellow
ENSG00000279267	AL078621.3	0.23269	turquoise
ENSG00000184730	APOBR	0.23222	brown
ENSG00000172572	PDE3A	0.23215	blue
ENSG00000105419	MEIS3	0.22922	turquoise
ENSG00000265688	MAFG-AS1	0.22683	yellow
ENSG00000144837	PLA1A	0.22175	brown
ENSG00000229807	XIST	0.21942	grey
ENSG00000236963	LINC01141	0.21719	turquoise
ENSG00000177464	GPR4	0.21254	brown
ENSG00000139971	C14orf37	0.21127	turquoise
ENSG00000196436	NPIPB15	0.20216	grey
ENSG00000237438	CECR7	0.19829	turquoise
ENSG00000232528	AL109809.1	0.1953	yellow
ENSG00000081052	COL4A4	0.19407	brown
ENSG00000236819	LINC01563	0.19138	turquoise
ENSG00000246982	Z84485.1	0.18968	grey
ENSG00000162992	NEUROD1	0.18426	turquoise
ENSG00000166573	GALR1	0.18271	turquoise
ENSG00000240583	AQP1	0.17767	blue
ENSG00000269707	AC018730.2	0.17621	turquoise
ENSG00000140961	OSGIN1	0.17176	grey
ENSG00000196812	ZSCAN16	0.17081	turquoise
ENSG00000204128	C2orf72	0.17066	turquoise
ENSG00000244734	HBB	0.1691	grey
ENSG00000141665	FBXO15	0.16457	turquoise
ENSG00000070731	ST6GALNAC2	0.16349	brown
ENSG00000118515	SGK1	0.16158	green
ENSG00000111181	SLC6A12	0.16093	green
ENSG00000013573	DDX11	0.16032	grey
ENSG00000163220	S100A9	0.15697	brown
ENSG00000183876	ARSI	0.15489	blue
ENSG00000102287	GABRE	0.15444	brown
ENSG00000099260	PALMD	0.15344	brown
ENSG00000233695	GAS6-AS1	0.14852	turquoise
ENSG00000143429	AC116050.1	0.14798	yellow
ENSG00000162551	ALPL	0.147	brown
ENSG00000125810	CD93	0.1433	brown
ENSG00000100376	FAM118A	0.1433	grey
ENSG00000240342	RPS2P5	0.14158	grey
ENSG00000226067	LINC00623	0.14089	turquoise
ENSG00000136982	DSCC1	0.14021	turquoise
ENSG00000155980	KIF5A	0.14008	blue
ENSG00000198624	CCDC69	0.13866	green
ENSG00000143318	CASQ1	0.13852	turquoise
ENSG00000176809	LRRC37A3	0.13851	turquoise
ENSG00000006611	USH1C	0.13791	blue
ENSG00000138336	TET1	0.13733	blue
ENSG00000110492	MDK	0.13713	turquoise
ENSG00000149418	ST14	0.13659	turquoise
ENSG00000056998	GYG2	0.13583	blue
ENSG00000198554	WDHD1	0.13521	turquoise
ENSG00000261609	GAN	0.13335	turquoise
ENSG00000224383	PRR29	0.13308	brown
ENSG00000126709	IFI6	0.13304	turquoise
ENSG00000154734	ADAMTS1	0.13281	brown
ENSG00000133466	C1QTNF6	0.13189	grey
ENSG00000272602	ZNF595	0.13104	turquoise
ENSG00000135269	TES	0.13042	brown
ENSG00000147509	RGS20	0.12943	blue
ENSG00000223865	HLA-DPB1	0.12734	brown
ENSG00000157379	DHRS1	0.12687	turquoise
ENSG00000260942	CAPN10-AS1	0.12653	grey
ENSG00000205517	RGL3	0.12633	brown
ENSG00000127325	BEST3	0.12603	blue
ENSG00000272690	LINC02018	0.12532	grey
ENSG00000160200	CBS	0.12513	grey
ENSG00000181019	NQO1	0.12456	blue
ENSG00000231768	LINC01354	0.12396	blue
ENSG00000188848	BEND4	0.12376	turquoise
ENSG00000146215	CRIP3	0.12269	turquoise
ENSG00000112964	GHR	0.12121	turquoise
ENSG00000162493	PDPN	0.11911	blue
ENSG00000140522	RLBP1	0.11496	blue
ENSG00000271254	AC240274.1	0.11495	turquoise
ENSG00000215559	ANKRD20A11P	0.11436	yellow
ENSG00000132832	AL139352.1	0.11323	turquoise
ENSG00000170379	TCAF2	0.11205	grey

ensembl ID	symbol	weight	module
ENSG00000267568	AC016168.2	0.11183	grey
ENSG00000242686	AC107464.1	0.11139	turquoise
ENSG00000114656	KIAA1257	0.11108	yellow
ENSG00000234327	AC012146.1	0.11097	turquoise
ENSG00000136859	ANGPTL2	0.11076	green
ENSG00000118523	CTGF	0.10811	brown
ENSG00000255769	GOLGA2P10	0.1075	turquoise
ENSG00000130287	NCAN	0.10637	blue
ENSG00000185864	NPIPB4	0.10473	turquoise
ENSG00000140807	NKD1	0.10394	green
ENSG00000188916	FAM196A	0.10389	turquoise
ENSG00000179477	ALOX12B	0.10367	turquoise
ENSG00000118777	ABCG2	0.10156	brown
ENSG00000205336	ADGRG1	0.10133	blue
ENSG00000087116	ADAMTS2	0.10058	turquoise
ENSG00000136826	KLF4	0.09994	brown
ENSG00000123454	DBH	0.099888	turquoise
ENSG00000137404	NRM	0.098639	brown
ENSG00000182109		0.098368	turquoise
ENSG00000121281	ADCY7	0.097449	turquoise
ENSG00000251442	LINC01094	0.096568	brown
ENSG00000273702	AC091271.1	0.096279	blue
ENSG00000107821	KAZALD1	0.095312	turquoise
ENSG00000129204	USP6	0.093962	turquoise
ENSG00000243244	STON1	0.093914	brown
ENSG00000233251	AC007743.1	0.093808	turquoise
ENSG00000270231	NBPF8	0.093277	turquoise
ENSG00000126249	PDCD2L	0.092782	turquoise
ENSG00000188039	NWD1	0.092777	green
ENSG00000119509	INVS	0.092774	turquoise
ENSG00000121858	TNFSF10	0.092424	grey
ENSG00000006534	ALDH3B1	0.090819	blue
ENSG00000099958	DERL3	0.090165	grey
ENSG00000104899	AMH	0.089358	grey
ENSG00000112210	RAB23	0.088158	turquoise
ENSG00000244026	FAM86DP	0.087718	turquoise
ENSG00000181631	P2RY13	0.087613	grey
ENSG00000112149	CD83	0.087075	turquoise
ENSG00000281501	SEPSECS-AS1	0.086068	yellow
ENSG00000254726	MEX3A	0.085666	grey
ENSG00000158406	HIST1H4H	0.08508	grey
ENSG00000227953	LINC01341	0.083835	turquoise
ENSG00000103742	IGDCC4	0.08377	blue
ENSG00000105707	HPN	0.083057	green
ENSG00000133069	TMCC2	0.082881	turquoise
ENSG00000156076	WIF1	0.0828	blue
ENSG00000198502	HLA-DRB5	0.082379	brown
ENSG00000232931	LINC00342	0.081125	turquoise
ENSG00000178796	RIIAD1	0.080697	turquoise
ENSG00000180481	GLIPR1L2	0.080023	grey
ENSG00000233058	LINC00884	0.079546	turquoise
ENSG00000171243	SOSTDC1	0.079334	turquoise
ENSG00000141179	PCTP	0.079217	turquoise
ENSG00000211448	DIO2	0.078864	grey
ENSG00000142621	FHAD1	0.078678	turquoise
ENSG00000163520	FBLN2	0.078269	turquoise
ENSG00000116830	TTF2	0.077922	turquoise
ENSG00000173918	C1QTNF1	0.077794	brown
ENSG00000182511	FES	0.076993	brown
ENSG00000175164	ABO	0.076358	brown
ENSG00000227827	AC138969.2	0.076133	turquoise
ENSG00000169246	NPIPB3	0.075304	turquoise
ENSG00000235072	AC012074.1	0.074836	turquoise

ensembl ID	symbol	weight	module
ENSG00000273142	AC073335.2	0.073886	grey
ENSG00000149150	SLC43A1	0.073885	turquoise
ENSG00000054392	HHAT	0.073682	turquoise
ENSG00000277494	GPIHBP1	0.072773	green
ENSG00000224914	LINC00863	0.072222	turquoise
ENSG00000260948	AL390195.2	0.07217	turquoise
ENSG00000186918	ZNF395	0.072135	blue
ENSG00000144057	ST6GAL2	0.071791	turquoise
ENSG00000162383	SLC1A7	0.071448	brown
ENSG00000162836	ACP6	0.070934	blue
ENSG00000188681	TEKT4P2	0.068776	grey
ENSG00000214425	LRRC37A4P	0.06871	yellow
ENSG00000173947	PIFO	0.068495	blue
ENSG00000072736	NFATC3	0.068112	turquoise
ENSG00000111962	UST	0.067421	turquoise
ENSG00000244480	AC005154.3	0.067129	turquoise
ENSG00000196154	S100A4	0.066933	green
ENSG00000148357	HMCN2	0.066764	turquoise
ENSG00000134323	MYCN	0.066344	turquoise
ENSG00000169418	NPR1	0.065934	brown
ENSG00000277758	FO681492.1	0.065003	turquoise
ENSG00000105675	ATP4A	0.064717	turquoise
ENSG00000267838	AC245884.8	0.06406	turquoise
ENSG00000141469	SLC14A1	0.061732	blue
ENSG00000255545	AP004608.1	0.061171	turquoise
ENSG00000006047	YBX2	0.060636	turquoise
ENSG00000185519	FAM131C	0.059725	turquoise
ENSG00000260426	AC008060.4	0.059616	turquoise
ENSG00000139675	HNRNPA1L2	0.059582	turquoise
ENSG00000196196	HRCT1	0.058657	grey
ENSG00000177551	NHLH2	0.058474	turquoise
ENSG00000077585	GPR137B	0.057915	blue
ENSG00000228716	DHFR	0.05731	grey
ENSG00000157833	GAREM2	0.056805	green
ENSG00000176641	RNF152	0.056721	brown
ENSG00000177425	PAWR	0.056696	brown
ENSG00000011426	ANLN	0.055581	green
ENSG00000016602	CLCA4	0.05436	green
ENSG00000173801	JUP	0.05383	blue
ENSG00000250903	GMDS-AS1	0.053526	turquoise
ENSG00000149809	TM7SF2	0.051891	turquoise
ENSG00000188536	HBA2	0.050951	yellow
ENSG00000144401	METTL21A	0.050115	turquoise
ENSG00000280670	CCDC163	0.049977	blue
ENSG00000196369	SRGAP2B	0.049672	turquoise
ENSG00000175643	RMI2	0.048871	turquoise
ENSG00000166473	PKD1L2	0.048246	green
ENSG00000081853	PCDHGA2	0.048241	blue
ENSG00000152527	PLEKHH2	0.047905	green
ENSG00000174807	CD248	0.047572	brown
ENSG00000171365	CLCN5	0.047279	turquoise
ENSG00000203883	SOX18	0.047181	yellow
ENSG00000242299	AC073861.1	0.046946	turquoise
ENSG00000253710	ALG11	0.046563	grey
ENSG00000117115	PADI2	0.046323	green
ENSG00000087245	MMP2	0.046031	brown
ENSG00000177076	ACER2	0.045974	turquoise
ENSG00000154764	WNT7A	0.045971	turquoise
ENSG00000179954	SSC5D	0.045913	turquoise
ENSG00000003400	CASP10	0.045252	brown
ENSG00000140545	MFGE8	0.044562	turquoise
ENSG00000167840	ZNF232	0.0444	turquoise
ENSG00000145014	TMEM44	0.04418	turquoise

ensembl ID	symbol	weight	module
ENSG00000120903	CHRNA2	0.04401	turquoise
ENSG00000122085	MTERF4	0.043004	turquoise
ENSG00000183379	SYNDIG1L	0.042898	turquoise
ENSG00000064666	CNN2	0.042205	brown
ENSG00000116711	PLA2G4A	0.04198	turquoise
ENSG00000241015	TPM3P9	0.041974	turquoise
ENSG00000225968	ELFN1	0.041724	turquoise
ENSG00000186960	LINC01551	0.041551	turquoise
ENSG00000225313	AL513327.1	0.041533	turquoise
ENSG00000270015	AC087481.3	0.041075	turquoise
ENSG00000165084	C8orf34	0.040337	turquoise
ENSG00000122557	HERPUD2	0.040103	turquoise
ENSG00000215440	NPEPL1	0.039073	grey
ENSG00000183506	PI4KAP2	0.039042	turquoise
ENSG00000201136	RNU6-353P	0.038874	yellow
ENSG00000151240	DIP2C	0.038573	turquoise
ENSG00000155254	MARVELD1	0.038509	turquoise
ENSG00000198821	CD247	0.038257	turquoise
ENSG00000103226	NOMO3	0.037798	turquoise
ENSG00000186231	KLHL32	0.037312	turquoise
ENSG00000122375	OPN4	0.037269	turquoise
ENSG00000196132	MYT1	0.037256	green
ENSG00000214756	CSKMT	0.036588	turquoise
ENSG00000166483	WEE1	0.036321	blue
ENSG00000104332	SFRP1	0.036097	turquoise
ENSG00000205918	PDPK2P	0.035848	turquoise
ENSG00000130055	GDPD2	0.035549	blue
ENSG00000271743	AF287957.1	0.035312	turquoise
ENSG00000125378	BMP4	0.03498	turquoise
ENSG00000115339	GALNT3	0.034953	turquoise
ENSG00000135097	MSI1	0.034616	blue
ENSG00000107562	CXCL12	0.034598	grey
ENSG00000261819	AC138932.3	0.034368	yellow
ENSG00000152208	GRID2	0.034281	grey
ENSG00000183496	MEX3B	0.034195	turquoise
ENSG00000110628	SLC22A18	0.033648	turquoise
ENSG00000172548	NIPAL4	0.033169	green
ENSG00000154721	JAM2	0.032771	blue
ENSG00000277702	AC239859.6	0.032637	turquoise
ENSG00000116874	WARS2	0.03253	turquoise
ENSG00000213096	ZNF254	0.032354	turquoise
ENSG00000134253	TRIM45	0.031874	turquoise
ENSG00000188234	AGAP4	0.031704	turquoise
ENSG00000225178	RPSAP58	0.031111	grey
ENSG00000250802	ZBED3-AS1	0.031101	blue
ENSG00000256269	HMBS	0.030967	turquoise
ENSG00000133519	ZDHHC8P1	0.030901	turquoise
ENSG00000230177	AL080317.1	0.030673	turquoise
ENSG00000079462	PAFAH1B3	0.030456	turquoise
ENSG00000227191	TRGC2	0.030352	grey
ENSG00000101883	RHOXF1	0.030191	grey
ENSG00000245910	SNHG6	0.029881	turquoise
ENSG00000259429	UBE2Q2P2	0.029878	yellow
ENSG00000179029	TMEM107	0.029857	turquoise
ENSG00000064300	NGFR	0.029709	brown
ENSG00000255198	SNHG9	0.029379	yellow
ENSG00000015133	CCDC88C	0.029173	turquoise
ENSG00000230202	AL450405.1	0.029077	yellow
ENSG00000224195	AC022400.1	0.028376	turquoise
ENSG00000215374	FAM66B	0.028089	turquoise
ENSG00000188290	HES4	0.02764	yellow
ENSG00000186891	TNFRSF18	0.027351	turquoise
ENSG00000282936	AC004706.4	0.027141	turquoise

ensembl ID	symbol	weight	module
ENSG00000102882	MAPK3	0.026837	turquoise
ENSG00000169908	TM4SF1	0.026577	brown
ENSG00000214189	ZNF788	0.026498	blue
ENSG00000167123	CERCAM	0.026382	green
ENSG00000171806	METTL18	0.02636	turquoise
ENSG00000272010	AC100814.1	0.026355	turquoise
ENSG00000180834	MAP6D1	0.026039	turquoise
ENSG00000229036	VDAC1P8	0.025499	turquoise
ENSG00000198826	ARHGAP11A	0.0252	turquoise
ENSG00000140750	ARHGAP17	0.025171	green
ENSG00000101224	CDC25B	0.024869	turquoise
ENSG00000149531	FRG1BP	0.024799	turquoise
ENSG00000157613	CREB3L1	0.024696	turquoise
ENSG00000095383	TBC1D2	0.024379	green
ENSG00000049283	EPN3	0.023883	turquoise
ENSG00000165092	ALDH1A1	0.02377	turquoise
ENSG00000113209	PCDHB5	0.023537	blue
ENSG00000225630	MTND2P28	0.023532	turquoise
ENSG00000255020	AF131216.3	0.023446	turquoise
ENSG00000131477	RAMP2	0.023316	grey
ENSG00000134899	ERCC5	0.023211	grey
ENSG00000127528	KLF2	0.023196	yellow
ENSG00000129993	CBFA2T3	0.022885	turquoise
ENSG00000071894	CPSF1	0.022546	turquoise
ENSG00000132275	RRP8	0.022522	turquoise
ENSG00000204172	AGAP9	0.02244	turquoise
ENSG00000162976	PQLC3	0.022311	turquoise
ENSG00000183091	NEB	0.021864	turquoise
ENSG00000167720	SRR	0.021825	turquoise
ENSG00000233901	LINC01503	0.021384	turquoise
ENSG00000272971	AL365181.4	0.021305	turquoise
ENSG00000105750	ZNF85	0.021183	turquoise
ENSG00000198547	C20orf203	0.020848	turquoise
ENSG00000105287	PRKD2	0.02078	grey
ENSG00000273748	AL592183.1	0.020534	grey
ENSG00000108375	RNF43	0.02045	blue
ENSG00000154262	ABCA6	0.020446	yellow
ENSG00000187678	SPRY4	0.020403	turquoise
ENSG00000277053	GTF2IP1	0.020253	turquoise
ENSG00000258702	AL137786.1	0.020096	turquoise
ENSG00000215769	ARHGAP27P1-BPTFP1-KPNA2P3	0.019563	turquoise
ENSG00000280187	AC022107.1	0.019505	turquoise
ENSG00000134207	SYT6	0.019458	turquoise
ENSG00000197993	KEL	0.019368	green
ENSG00000141314	RHBDL3	0.019215	turquoise
ENSG00000172301	COPRS	0.019208	turquoise
ENSG00000188242	AC010442.1	0.019108	turquoise
ENSG00000258010	AC016705.1	0.018708	turquoise
ENSG00000120071	KANSL1	0.01824	turquoise
ENSG00000261126	RBFADN	0.017896	turquoise
ENSG00000162745	OLFML2B	0.01788	turquoise
ENSG00000115318	LOXL3	0.017866	blue
ENSG00000280255	AC004947.2	0.01785	turquoise
ENSG00000177335	C8orf31	0.0178	turquoise
ENSG00000134020	PEBP4	0.017625	turquoise
ENSG00000261377	PDCD6IPP2	0.017538	turquoise
ENSG00000157978	LDLRAP1	0.017277	green
ENSG00000272419	AC241585.2	0.017195	turquoise
ENSG00000155792	DEPTOR	0.017184	turquoise
ENSG00000153495	TEX29	0.017141	turquoise

ensembl ID	symbol	weight	module
ENSG00000129534	MIS18BP1	0.016773	turquoise
ENSG00000066294	CD84	0.016549	brown
ENSG00000261386	AC027682.4	0.016451	turquoise
ENSG00000183921	SDR42E2	0.016247	turquoise
ENSG00000259495	AC016705.2	0.016217	turquoise
ENSG00000169857	AVEN	0.016122	turquoise
ENSG00000135046	ANXA1	0.016084	brown
ENSG00000139352	ASCL1	0.015893	green
ENSG00000103995	CEP152	0.015656	yellow
ENSG00000204611	ZNF616	0.015592	turquoise
ENSG00000154263	ABCA10	0.015493	turquoise
ENSG00000242732	RTL5	0.01514	turquoise
ENSG00000088053	GP6	0.014677	turquoise
ENSG00000136319	TTC5	0.014085	turquoise
ENSG00000215915	ATAD3C	0.013768	turquoise
ENSG00000161940	BCL6B	0.013534	brown
ENSG00000173163	COMMD1	0.013398	turquoise
ENSG00000222044	AL031587.1	0.013157	yellow
ENSG00000261353		0.012984	grey
ENSG00000280351	AC127496.7	0.012956	yellow
ENSG00000204323	SMIM5	0.012679	green
ENSG00000078295	ADCY2	0.012572	blue
ENSG00000125850	OVOL2	0.012543	turquoise
ENSG00000272668	AL590560.2	0.012513	grey
ENSG00000147571	CRH	0.012283	turquoise
ENSG00000206344	HCG27	0.012264	yellow
ENSG00000185269	NOTUM	0.012222	turquoise
ENSG00000112773	FAM46A	0.012208	brown
ENSG00000138942	RNF185	0.011899	turquoise
ENSG00000268751	SCGB1B2P	0.011876	yellow
ENSG00000242220	TCP10L	0.011647	turquoise
ENSG00000173653	RCE1	0.011606	turquoise
ENSG00000279656	AL132780.4	0.011584	grey
ENSG00000164904	ALDH7A1	0.011286	blue
ENSG00000064787	BCAS1	0.011277	turquoise
ENSG00000261373	VPS9D1-AS1	0.011161	yellow
ENSG00000101871	MID1	0.011119	blue
ENSG00000248049	UBA6-AS1	0.011014	turquoise
ENSG00000269935	AC092720.2	0.010962	turquoise
ENSG00000123689	G0S2	0.010793	grey
ENSG00000273308	AC024560.3	0.010598	yellow
ENSG00000138741	TRPC3	0.010503	turquoise
ENSG00000165553	NGB	0.010303	turquoise
ENSG00000266338	NBPF15	0.010285	turquoise
ENSG00000132837	DMGDH	0.010225	turquoise
ENSG00000254081	LINC01299	0.010091	grey
ENSG00000065268	WDR18	0.0099383	turquoise
ENSG00000133433	GSTT2B	0.0097166	turquoise
ENSG00000114779	ABHD14B	0.0094285	turquoise
ENSG00000220804	LINC01881	0.0093006	yellow
ENSG00000101282	RSPO4	0.0089581	turquoise
ENSG00000243742	RPLP0P2	0.0086551	grey
ENSG00000144283	PKP4	0.0081785	turquoise
ENSG00000111364	DDX55	0.0078924	yellow
ENSG00000185904	LINC00839	0.0078883	turquoise
ENSG00000136114	THSD1	0.0078444	blue
ENSG00000220008	LINGO3	0.0077896	yellow
ENSG00000227782	AC002553.1	0.0075723	yellow
ENSG00000118514	ALDH8A1	0.0074187	turquoise
ENSG00000203867	RBM20	0.007079	turquoise
ENSG00000108370	RGS9	0.006996	blue
ENSG00000143994	ABHD1	0.0069629	blue
ENSG00000251429	AC098679.2	0.0069347	green

ensembl ID	symbol	weight	module
ENSG00000101311	FERMT1	0.0069089	green
ENSG00000167874	TMEM88	0.0068412	grey
ENSG00000075461	CACNG4	0.0067956	green
ENSG00000164818	DNAAF5	0.0067319	turquoise
ENSG00000181773	GPR3	0.0066865	turquoise
ENSG00000136147	PHF11	0.0066154	turquoise
ENSG00000267280	TBX2-AS1	0.006453	brown
ENSG00000198938	MT-CO3	0.0064236	turquoise
ENSG00000120279	MYCT1	0.0063932	brown
ENSG00000160999	SH2B2	0.0063499	yellow
ENSG00000260025	AC009414.2	0.006246	turquoise
ENSG00000280485	AL358154.1	0.0061272	yellow
ENSG00000182853	VMO1	0.0060622	turquoise
ENSG00000140795	MYLK3	0.006018	turquoise
ENSG00000173457	PPP1R14B	0.0058658	turquoise
ENSG00000172985	SH3RF3	0.005857	turquoise
ENSG00000214491	SEC14L6	0.0058494	turquoise
ENSG00000103485	QPRT	0.0058033	turquoise
ENSG00000028116	VRK2	0.0055534	green
ENSG00000171094	ALK	0.0052428	turquoise
ENSG00000197536	C5orf56	0.0052398	turquoise
ENSG00000272674	PCDHB16	0.0051392	turquoise
ENSG00000270728	AL035413.2	0.0051353	yellow
ENSG00000144061	NPHP1	0.004951	turquoise
ENSG00000177984	LCN15	0.004577	turquoise
ENSG00000102390	PBDC1	0.0045051	turquoise
ENSG00000132793	LPIN3	0.0043651	blue
ENSG00000255836	AC131206.1	0.0042159	yellow
ENSG00000166086	JAM3	0.0041743	turquoise
ENSG00000272512	AL645608.8	0.0041256	grey
ENSG00000238197	PAXBP1-AS1	0.0039422	turquoise
ENSG00000135824	RGS8	0.0037904	turquoise
ENSG00000163485	ADORA1	0.0037369	turquoise
ENSG00000175768	TOMM5	0.0035518	turquoise
ENSG00000280734	LINC01232	0.0034469	turquoise
ENSG00000170684	ZNF296	0.0034448	yellow
ENSG00000132846	ZBED3	0.0034333	green
ENSG00000198468	FLVCR1-AS1	0.0032206	grey
ENSG00000183066	WBP2NL	0.0030944	yellow
ENSG00000137269	LRRC1	0.0030549	green
ENSG00000169064	ZBBX	0.0029782	turquoise
ENSG00000198727	MT-CYB	0.002951	turquoise
ENSG00000140022	STON2	0.0028454	blue
ENSG00000196502	SULT1A1	0.0028402	turquoise
ENSG00000260912	AL158206.1	0.0028194	yellow
ENSG00000267107	PCAT19	0.0028068	brown
ENSG00000170011	MYRIP	0.0027228	turquoise
ENSG00000196735	HLA-DQA1	0.0026605	brown
ENSG00000198886	MT-ND4	0.0026325	turquoise
ENSG00000196418	ZNF124	0.0026246	yellow
ENSG00000205744	DENND1C	0.0026099	brown
ENSG00000137766	UNC13C	0.0026098	turquoise
ENSG00000125804	FAM182A	0.0025733	grey
ENSG00000146834	MEPCE	0.0025464	turquoise
ENSG00000180354	MTURN	0.002539	turquoise
ENSG00000171388	APLN	0.0024605	green
ENSG00000228543	AC003684.1	0.0024508	grey
ENSG00000187775	DNAH17	0.0024143	green
ENSG00000152133	GPATCH11	0.0023659	turquoise
ENSG00000189134	NKAPL	0.0022865	yellow
ENSG00000129467	ADCY4	0.0022664	brown
ENSG00000215252	GOLGA8B	0.0022564	turquoise
ENSG00000100439	ABHD4	0.0022178	blue

ensembl ID	symbol	weight	module
ENSG00000188803	SHISA6	0.0021551	blue
ENSG00000140577	CRTC3	0.0020721	turquoise
ENSG00000163046	ANKRD30BL	0.0020595	yellow
ENSG00000228451	SDAD1P1	0.002031	grey
ENSG00000069122	ADGRF5	0.0020031	brown
ENSG00000124508	BTN2A2	0.0019836	blue
ENSG00000091986	CCDC80	0.0019355	blue
ENSG00000181026	AEN	0.0018551	brown
ENSG00000230373	GOLGA6L5P	0.0018539	yellow
ENSG00000216775	AL109918.1	0.0017677	turquoise
ENSG00000084628	NKAIN1	0.0017654	turquoise
ENSG00000159433	STARD9	0.0016761	turquoise
ENSG00000130518	IQCN	0.0015905	turquoise
ENSG00000167771	RCOR2	0.0015789	turquoise
ENSG00000198804	MT-CO1	0.0015404	turquoise
ENSG00000144115	THNSL2	0.0015386	grey
ENSG00000169629	RGPD8	0.0015323	turquoise
ENSG00000186188	FFAR4	0.0014579	turquoise
ENSG00000261810	AC133565.1	0.0014515	yellow
ENSG00000180777	ANKRD30B	0.0014506	yellow
ENSG00000212907	MT-ND4L	0.0013873	turquoise
ENSG00000281708	ERC2-IT1	0.0013602	yellow
ENSG00000255495	AC145124.1	0.0013126	yellow
ENSG00000197061	HIST1H4C	0.0012951	yellow
ENSG00000231365	AL359915.2	0.0012533	turquoise
ENSG00000204219	TCEA3	0.0012253	blue
ENSG00000179455	MKRN3	0.0012236	green
ENSG00000282508	LINC01002	0.0011844	turquoise
ENSG00000198546	ZNF511	0.0011379	grey
ENSG00000087086	FTL	0.0011253	blue
ENSG00000188786	MTF1	0.001069	turquoise
ENSG00000144824	PHLDB2	0.0010652	turquoise
ENSG00000243051	RN7SL269P	0.001046	yellow
ENSG00000136111	TBC1D4	0.0010354	turquoise
ENSG00000085276	MECOM	0.0010352	brown
ENSG00000145002	FAM86B2	0.00098662	turquoise
ENSG00000188783	PRELP	0.00098385	green
ENSG00000210082	MT-RNR2	0.00097678	turquoise
ENSG00000048540	LMO3	0.0009693	turquoise
ENSG00000087077	TRIP6	0.00092652	blue
ENSG00000123143	PKN1	0.00087972	blue
ENSG00000126217	MCF2L	0.00084918	turquoise
ENSG00000254838	GVINP1	0.00084161	yellow
ENSG00000064703	DDX20	0.00083476	turquoise
ENSG00000115970	THADA	0.00080497	turquoise
ENSG00000081479	LRP2	0.00077533	green
ENSG00000256193	LINC00507	0.00077061	turquoise
ENSG00000102057	KCND1	0.00074378	turquoise
ENSG00000128564	VGF	0.00073085	turquoise
ENSG00000264920	AC018521.5	0.00070618	turquoise
ENSG00000145721	LIX1	0.00069437	blue
ENSG00000189056	RELN	0.00066443	turquoise
ENSG00000187398	LUZP2	0.00063733	turquoise
ENSG00000274020	LINC01138	0.00061745	blue
ENSG00000274642	AC244669.2	0.00061283	turquoise
ENSG00000168754	FAM178B	0.00061088	grey
ENSG00000005961	ITGA2B	0.00056254	turquoise
ENSG00000148824	MTG1	0.00054822	turquoise
ENSG00000189376	C8orf76	0.00054639	turquoise
ENSG00000124201	ZNFX1	0.00054639	turquoise
ENSG00000177666	PNPLA2	0.00054162	turquoise
ENSG00000101187	SLCO4A1	0.00053347	brown
ENSG00000204685	STARD7-AS1	0.00051409	turquoise
ENSG00000197958	RPL12	0.00050554	turquoise
ENSG00000153066	TXNDC11	0.00044926	turquoise
ENSG00000160293	VAV2	0.00044486	turquoise
ENSG00000232388	SMIM26	0.00044214	turquoise
ENSG00000122547	EEPD1	0.00043752	turquoise
ENSG00000204392	LSM2	0.00042365	turquoise
ENSG00000177025	C19orf18	0.00038519	grey
ENSG00000125637	PSD4	0.00038495	blue
ENSG00000157399	ARSE	0.00038198	blue
ENSG00000255182	AC084125.2	0.00036771	turquoise
ENSG00000171105	INSR	0.00036376	green
ENSG00000137310	TCF19	0.00035297	brown
ENSG00000162623	TYW3	0.0003524	turquoise
ENSG00000132357	CARD6	0.00034801	brown
ENSG00000113721	PDGFRB	0.0003364	green
ENSG00000087087	SRRT	0.00032546	turquoise
ENSG00000198763	MT-ND2	0.00031138	turquoise
ENSG00000187867	PALM3	0.00029042	turquoise
ENSG00000242193	CRYZL2P	0.00027308	turquoise
ENSG00000092295	TGM1	0.00026996	turquoise
ENSG00000102935	ZNF423	0.00026269	blue
ENSG00000250067	YJEFN3	0.00025408	turquoise
ENSG00000279289	AL136164.3	0.00023655	yellow
ENSG00000166428	PLD4	0.0002263	grey
ENSG00000033867	SLC4A7	0.00021756	yellow
ENSG00000265218	AC103810.2	0.00021457	yellow
ENSG00000100814	CCNB1IP1	0.00021311	turquoise
ENSG00000229956	ZRANB2-AS2	0.00018135	yellow
ENSG00000166763	STRCP1	0.00017843	turquoise
ENSG00000142227	EMP3	0.00017697	brown
ENSG00000146535	GNA12	0.00017026	blue
ENSG00000266644	AC103810.5	0.00016552	yellow
ENSG00000275620	AL121827.2	0.00016073	blue

Supplemental Table 8 Model metrics for the linear regression between global cognitive function and the dependent variables stratified by SI modules in ROSMAP DLPFC samples. Cells with significant p values (< 0.05) are shown in bold.

target = global cognitive function	blue module						brown module						green module					
	Estimate	Std. Error	t value	Pr(>|t|)	PVE	sig. code	Estimate	Std. Error	t value	Pr(>|t|)	PVE	sig. code	Estimate	Std. Error	t value	Pr(>|t|)	PVE	sig. code
(Intercept)	-0.176	0.874	-0.202	8.40E-01			-0.310	0.898	-0.345	7.30E-01			-0.526	0.832	-0.632	5.27E-01		
SI	-0.045	0.009	-5.065	**6.23E-07**	10.45	***	-0.023	0.008	-2.826	**4.94E-03**	2.34	**	-0.054	0.007	-7.923	**2.28E-14**	21.10	***
age_death	-0.023	0.007	-3.055	**2.40E-03**	3.01	**	-0.030	0.007	-4.036	**6.51E-05**	5.91	***	-0.021	0.007	-2.982	**3.04E-03**	2.82	**
educ	0.034	0.014	2.468	**1.40E-02**	0.15	*	0.035	0.014	2.532	**1.17E-02**	0.29	*	0.034	0.013	2.614	**9.29E-03**	0.26	**
msex	-0.043	0.091	-0.470	6.39E-01	0.20		-0.022	0.094	-0.232	8.17E-01	0.12		-0.004	0.088	-0.040	9.68E-01	0.01	
race7	-0.272	0.336	-0.809	4.19E-01	0.28		-0.325	0.343	-0.949	3.43E-01	0.32		-0.110	0.323	-0.340	7.34E-01	0.04	
apoe4	-0.463	0.093	-4.989	**9.04E-07**	6.05	***	-0.530	0.094	-5.620	**3.57E-08**	8.19	***	-0.374	0.090	-4.146	**4.13E-05**	3.86	***
RIN	0.073	0.045	1.608	1.09E-01	0.32		0.120	0.045	2.656	**8.21E-03**	1.14	**	0.121	0.042	2.860	**4.45E-03**	1.23	**
PMI	0.021	0.010	2.102	**3.61E-02**	0.37	*	0.019	0.010	1.855	6.43E-02	0.26	.	0.010	0.009	1.097	2.73E-01	0.02	
r_pd	0.303	0.058	5.241	**2.58E-07**	6.23	***	0.290	0.059	4.879	**1.54E-06**	5.70	***	0.319	0.055	5.743	**1.83E-08**	6.43	***
r_stroke	0.113	0.065	1.728	8.47E-02	0.30	.	0.118	0.067	1.775	7.66E-02	0.33	.	0.099	0.063	1.578	1.15E-01	0.23	
dlbdx3	-0.756	0.146	-5.188	**3.38E-07**	4.44	***	-0.749	0.149	-5.028	**7.48E-07**	4.45	***	-0.557	0.142	-3.924	**1.02E-04**	2.28	***
hspath_typ	-0.744	0.186	-4.004	**7.41E-05**	2.62	***	-0.747	0.190	-3.938	**9.69E-05**	2.64	***	-0.587	0.179	-3.271	**1.16E-03**	1.60	**
arteriol_scler	-0.083	0.043	-1.947	5.23E-02	0.61	.	-0.079	0.044	-1.801	7.25E-02	0.55	.	-0.061	0.041	-1.493	1.36E-01	0.33	
Multiple R^2	0.350						0.322						0.402					
Adjusted R^2	0.326						0.297						0.380					

target = global cognitive function	grey module						turquoise module						yellow module					
	Estimate	Std. Error	t value	Pr(>|t|)	PVE	sig. code	Estimate	Std. Error	t value	Pr(>|t|)	PVE	sig. code	Estimate	Std. Error	t value	Pr(>|t|)	PVE	sig. code
(Intercept)	-1.223	0.903	-1.355	1.76E-01			1.481	0.840	1.764	7.85E-02		.	0.512	1.142	0.449	6.54E-01		
SI	0.040	0.014	2.810	**5.20E-03**	3.52	**	-0.057	0.006	-9.565	**< 2E-16**	23.65	***	-0.058	0.034	-1.734	8.36E-02	2.85	.
age_death	-0.032	0.007	-4.399	**1.39E-05**	5.40	***	-0.020	0.007	-3.008	**2.79E-03**	1.93	**	-0.029	0.007	-3.932	**9.92E-05**	5.02	***
educ	0.032	0.014	2.266	**2.40E-02**	0.05	*	0.036	0.013	2.869	**4.34E-03**	0.13	**	0.031	0.014	2.208	**2.78E-02**	0.07	*
msex	-0.063	0.093	-0.671	5.03E-01	0.45		-0.173	0.086	-2.015	**4.46E-02**	0.89	*	-0.048	0.094	-0.507	6.13E-01	0.22	
race7	-0.353	0.343	-1.030	3.04E-01	0.18		-0.198	0.313	-0.633	5.27E-01	0.47		-0.289	0.346	-0.835	4.04E-01	0.30	
apoe4	-0.525	0.094	-5.569	**4.68E-08**	7.55	***	-0.435	0.086	-5.037	**7.13E-07**	4.67	***	-0.513	0.095	-5.419	**1.03E-07**	7.54	***
RIN	0.095	0.046	2.065	**3.96E-02**	1.31	*	-0.144	0.049	-2.911	**3.80E-03**	2.41	**	0.096	0.047	2.041	**4.19E-02**	0.54	*
PMI	0.025	0.010	2.430	1.55E-02	0.27	*	0.015	0.009	1.598	1.11E-01	0.15		0.021	0.010	2.068	**3.93E-02**	0.40	*
r_pd	0.289	0.060	4.858	**1.70E-06**	6.26	***	0.272	0.054	5.037	**7.15E-07**	4.73	***	0.309	0.059	5.200	**3.17E-07**	6.53	***
r_stroke	0.123	0.067	1.849	6.52E-02	0.46	.	0.117	0.061	1.923	5.52E-02	0.36	.	0.125	0.067	1.873	6.18E-02	0.38	.
dlbdx3	-0.751	0.149	-5.045	**6.86E-07**	4.31	***	-0.526	0.138	-3.820	**1.55E-04**	2.17	***	-0.740	0.150	-4.931	**1.20E-06**	4.38	***
hspath_typ	-0.780	0.190	-4.103	**4.94E-05**	2.30	***	-0.630	0.173	-3.630	**3.20E-04**	1.86	***	-0.734	0.191	-3.841	**1.43E-04**	2.56	***
arteriol_scler	-0.077	0.044	-1.767	7.81E-02	0.41	.	-0.055	0.040	-1.382	1.68E-01	0.27		-0.085	0.044	-1.931	5.42E-02	0.63	.
Multiple R^2	0.322						0.437						0.314					
Adjusted R^2	0.297						0.416						0.289					

Signif. codes: 0 ‘***’ 0.001 ‘**’ 0.01 ‘*’ 0.05 ‘.’ 0.1 ‘ ’ 1
