# Supplementary material for: Deep learning-based brain transcriptomic signatures associated with the neuropathological and clinical severity of Alzheimer’s disease
Source: Brain Commun. 2021 Dec 14;4(1):fcab293. doi: 10.1093/braincomms/fcab293 (PMC8728025; doi:10.1093/braincomms/fcab293)
Supplement: fcab293_Supplementary_Data [file fcab293_supplementary_data.zip › Supplementary material_12022021.docx]

**Supplementary material**

**Supplementary Supplemental Figure legends**

**Supplemental Figure 1 Hyper-parameter estimation and model training. (A)** The regularization parameter λ was estimated to be 0.004 based on the one-standard-error rule. **(B)** The trade-off parameter α was estimated to be 2. (**C)** Curves of training and validation losses vs. epochs. No sign of over-fitting was observed. The first 1500 epochs are for model initialization based on supervised learning and the remaining 5000 epochs are for model optimization. (**D)** Curves of training and validation accuracy vs. epochs.

**Supplemental Figure 2 Index gene identification.** **(A)** Histogram of the weights in logarithm scale for each gene in the training model. (**B)** Volcano plot showing the distribution of the index genes vs all the other genes in ROSMAP dataset. P and log_2_FC values were obtained from DEG analysis (syn8456629). (**C)** Volcano plot showing the distribution of the index genes vs all the DEGs identified in the meta-analysis of AMP-AD datasets (syn11914606). P and log_2_FC values were the same as (**B**), taken from ROSMAP dataset (syn8456629), but genes not identified as DEGs in the meta-analysis are not shown for clarity.

**Supplemental Figure 3 Regression plots between SI and global cognitive function for the index genes from each module. (A)** blue; **(B)** brown; **(C)** green; **(D)** grey; (**E)** turquoise; **(F)** yellow.

**Supplemental Figure 4 Regression plots between SI and global cognitive function for the stratified groups by diagnosis.** **(A)** All AD and control subjects (used in supervised model training); **(B)** All other subjects (not used in supervised model training). The fitting lines for all subjects (bold) and stratified group (grey) are also shown.

**Supplemental Figure 5 The SI modules were examined for overlap (Fisher’s exact test) with the curated AD gene sets and co-expression modules derived from the individual dataset of AMP-AD cohorts.** Overlaps were shown for those adjusted p (FDR correction) < 0.05. Correction was done independently for each set or each study. **(A)** The curated gene sets were taken from Genecards,^1^ GeneRIF,^2^ Panther,^3^ dbGaP,^4^ IGAP,^5^ OMIM,^6^ KEGG,^7^ and WikiPathway^8^ respectively. The module genes were taken from **(B)** Mayo network^9^, **(C)** MSBB network^10^, **(D)** ROSMAP network^11^. Only those modules reported as associated and/or top ranked with AD traits were included in Supplemental Figure (**B-D)**.

**Supplemental Figure 6 Comparisons between SI and pseudotime from the work of Mukherjee et al. obtained on the same subjects (n = 537) in the ROSMAP cohort. (A)** Correlation plots between SI and pseudotime in female and male samples respectively. Correlation coefficients were obtained by lm function in R, with no covariates included. (**B)** Distribution of samples’ SI for three different external measures of AD staging: Braak score (tau pathology), CERAD score (amyloid pathology), and cognitive diagnosis (clinical measure of disease severity), stratified by sex (in comparison with Supplemental Figure 3 and S9 in Mukherjee et al). (**C)** Association p-values between SI/pseudotime and the three external measures, by linear or logistic regression, stratified by sex.

**Supplemental Figure S7 Cell-type gene expression signatures as a function of SI. Mean expression of cell markers for astrocytes, neurons, microglia, oligodendrocytes and endothelial cells were plotted and colored respectively. (A)** all subjects. **(B)** female only. **(C)** male only.

**Supplementary Table legends**

**Supplemental Table 1 Demographic information for all the subjects included in RNA-seq data used in this study.**

**Supplemental Table 2 Description of the variables used in the linear regression model for ROSMAP cohort.**

**Supplemental Table 3 Model metrics for the linear regression between global cognitive function and the dependent variables (as defined in Supplemental Table 2) stratified by diagnosis groups in ROSMAP DLPFC samples.**

**Supplemental Table 4 Model metrics (p values and correlation coefficients) for the linear regression between all the neuropathological biomarkers and the dependent variables (as defined in Supplemental Table 2) stratified by diagnosis groups in ROSMAP DLPFC samples.**

**Supplemental Table 5 Model metrics (p values and correlation coefficients) for the linear regression between neuropathological biomarkers and the dependent variables for the two brain regions in Mayo samples.**

**Supplemental Table 6 Model metrics (p values and correlation coefficients) for the linear regression between neuropathological and clinical biomarkers and the dependent variables for the four brain regions in MSBB samples.**

**Supplemental Table 7 Index genes and their weights contributing to the deep learning model.**

**Supplemental Table 8 Model metrics for the linear regression between global cognitive function and the dependent variables stratified by SI modules in ROSMAP DLPFC samples.**

**Supplemental references**

1. Stelzer G, Rosen N, Plaschkes I, et al. The GeneCards Suite: From Gene Data Mining to Disease Genome Sequence Analyses. *Curr Protoc Bioinformatics.* 2016;54:1 30 31-31 30 33.

2. Lu Z, Cohen KB, Hunter L. Finding Generifs Via Gene Ontology Annotations. Biocomputing 2006; 2005.

3. Mi H, Ebert D, Muruganujan A, et al. PANTHER version 16: a revised family classification, tree-based classification tool, enhancer regions and extensive API. *Nucleic Acids Res.* 2021;49(D1):D394-D403.

4. Tryka KA, Hao L, Sturcke A, et al. NCBI's Database of Genotypes and Phenotypes: dbGaP. *Nucleic Acids Res.* 2014;42(Database issue):D975-979.

5. Kunkle BW, Grenier-Boley B, Sims R, et al. Genetic meta-analysis of diagnosed Alzheimer's disease identifies new risk loci and implicates Abeta, tau, immunity and lipid processing. *Nat Genet.* 2019;51(3):414-430.

6. Amberger JS, Bocchini CA, Scott AF, Hamosh A. OMIM.org: leveraging knowledge across phenotype-gene relationships. *Nucleic Acids Res.* 2019;47(D1):D1038-D1043.

7. Kanehisa M, Furumichi M, Sato Y, Ishiguro-Watanabe M, Tanabe M. KEGG: integrating viruses and cellular organisms. *Nucleic Acids Res.* 2021;49(D1):D545-D551.

8. Martens M, Ammar A, Riutta A, et al. WikiPathways: connecting communities. *Nucleic Acids Res.* 2021;49(D1):D613-D621.

9. Allen M, Wang X, Burgess JD, et al. Conserved brain myelination networks are altered in Alzheimer's and other neurodegenerative diseases. *Alzheimers Dement.* 2018;14(3):352-366.

10. Wang M, Roussos P, McKenzie A, et al. Integrative network analysis of nineteen brain regions identifies molecular signatures and networks underlying selective regional vulnerability to Alzheimer's disease. *Genome Med.* 2016;8(1):104.

11. Mostafavi S, Gaiteri C, Sullivan SE, et al. A molecular network of the aging human brain provides insights into the pathology and cognitive decline of Alzheimer's disease. *Nat Neurosci.* 2018;21(6):811-819.
