# Supplementary material for: Deep learning-based brain transcriptomic signatures associated with the neuropathological and clinical severity of Alzheimer’s disease
Source: Brain Commun. 2021 Dec 14;4(1):fcab293. doi: 10.1093/braincomms/fcab293 (PMC8728025; doi:10.1093/braincomms/fcab293)
Supplement: fcab293_Supplementary_Data [file fcab293_supplementary_data.zip › SupplementalFigures.pdf]

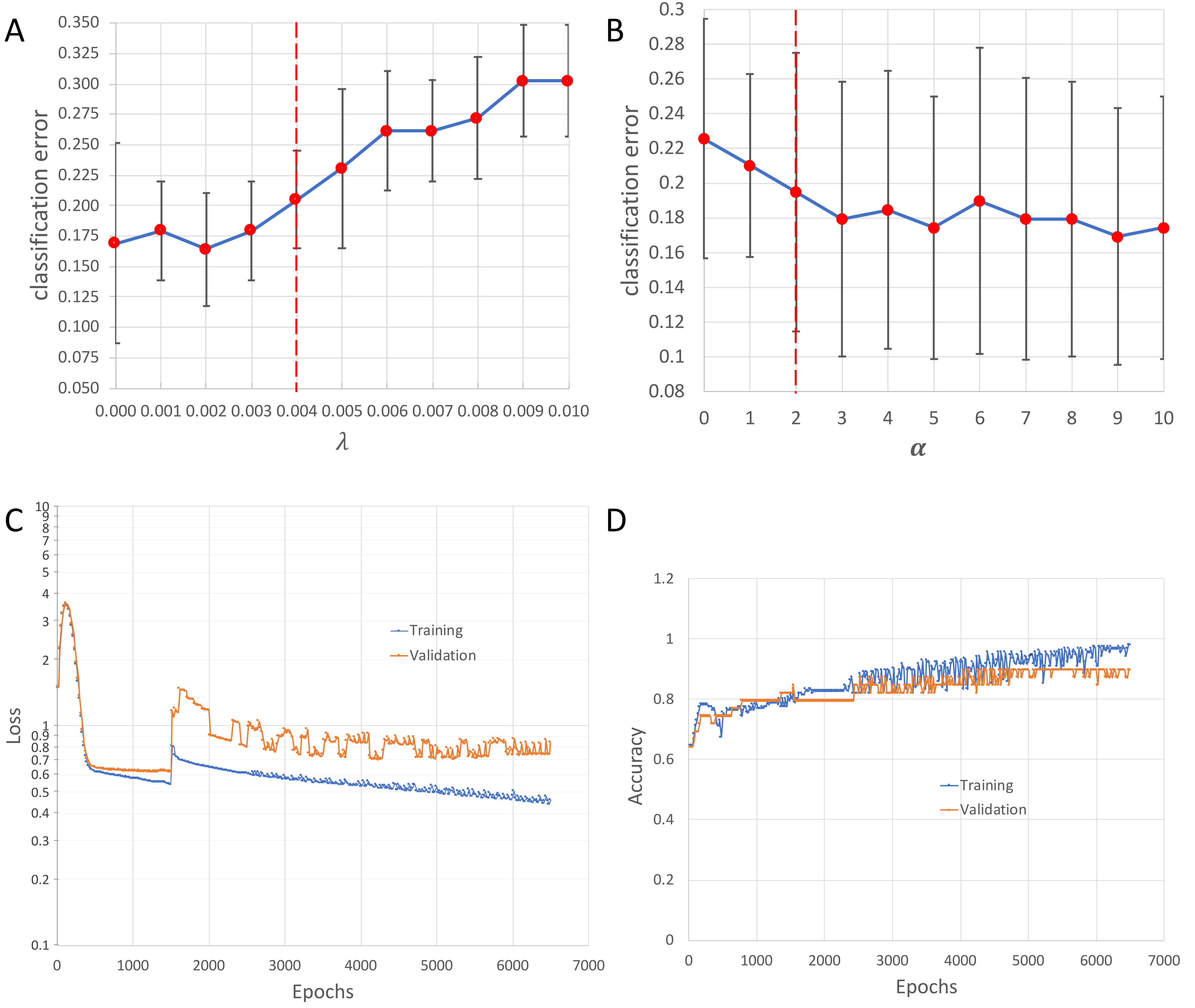

Supplementary Figure 1

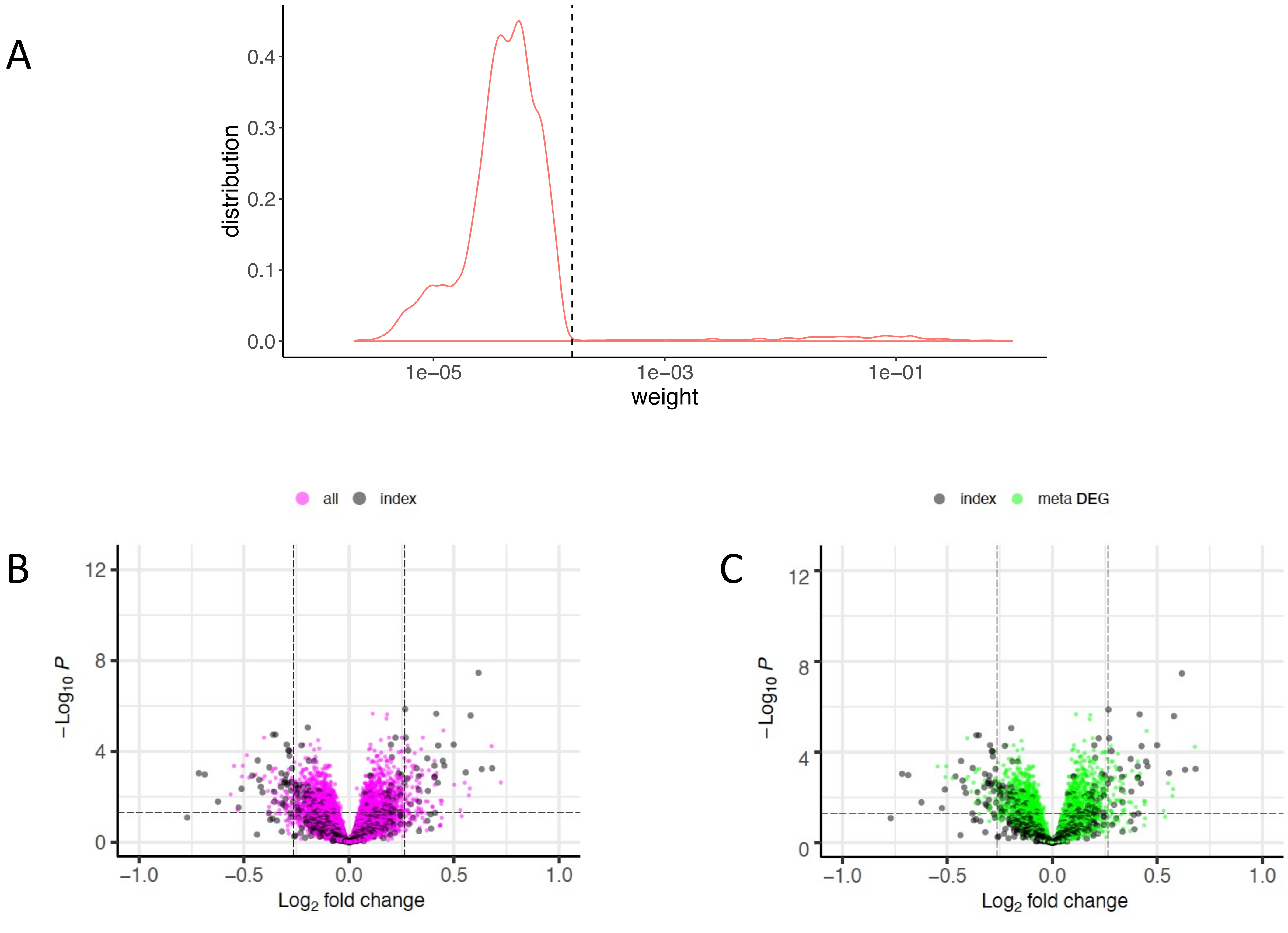

Supplementary Figure 2

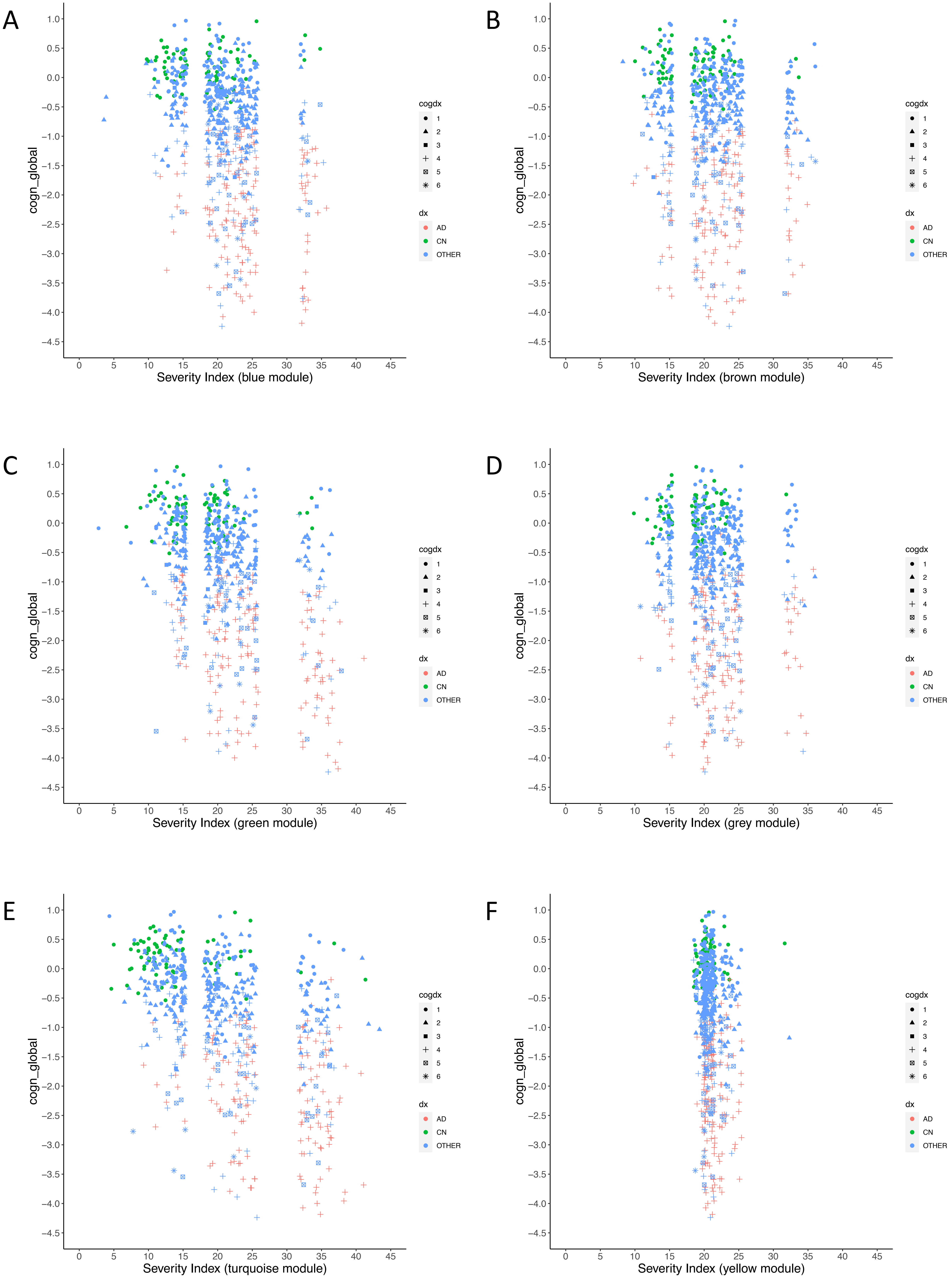

Supplementary Figure 3

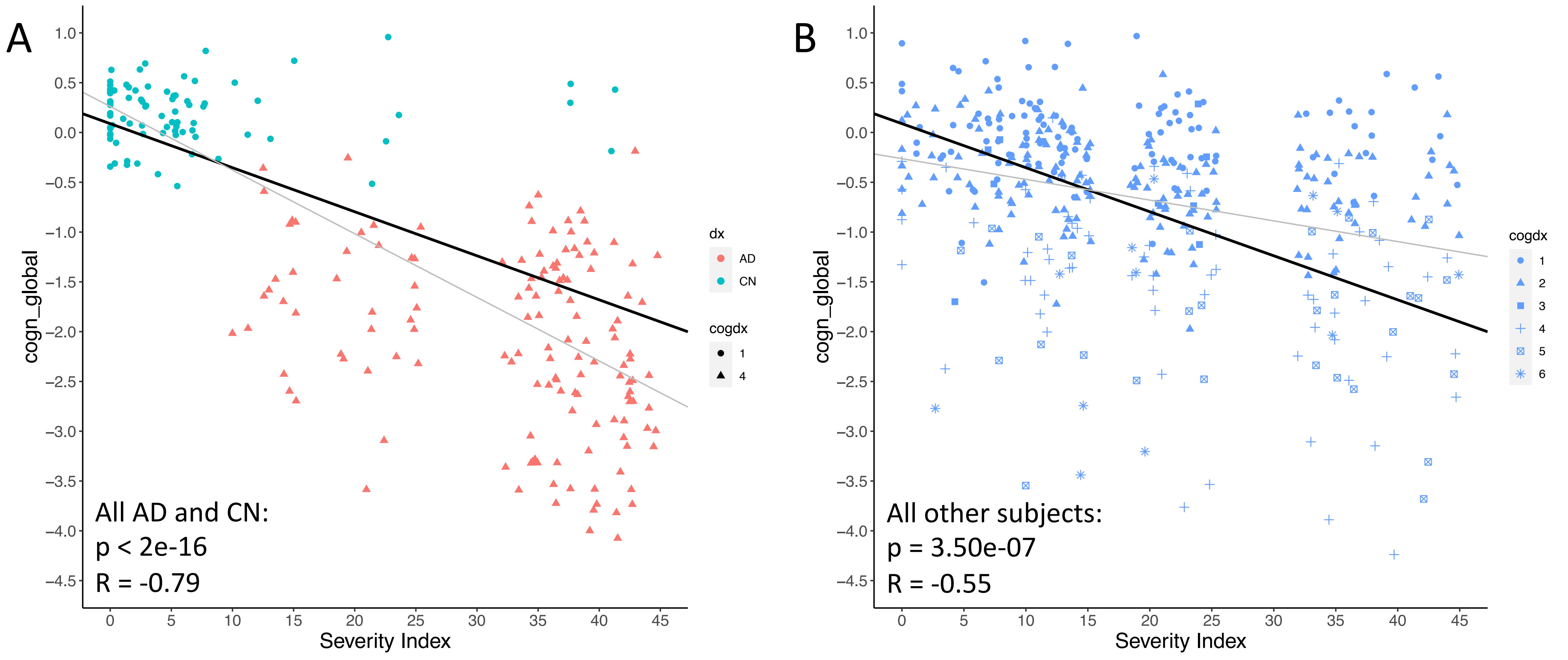

Supplementary Figure 4

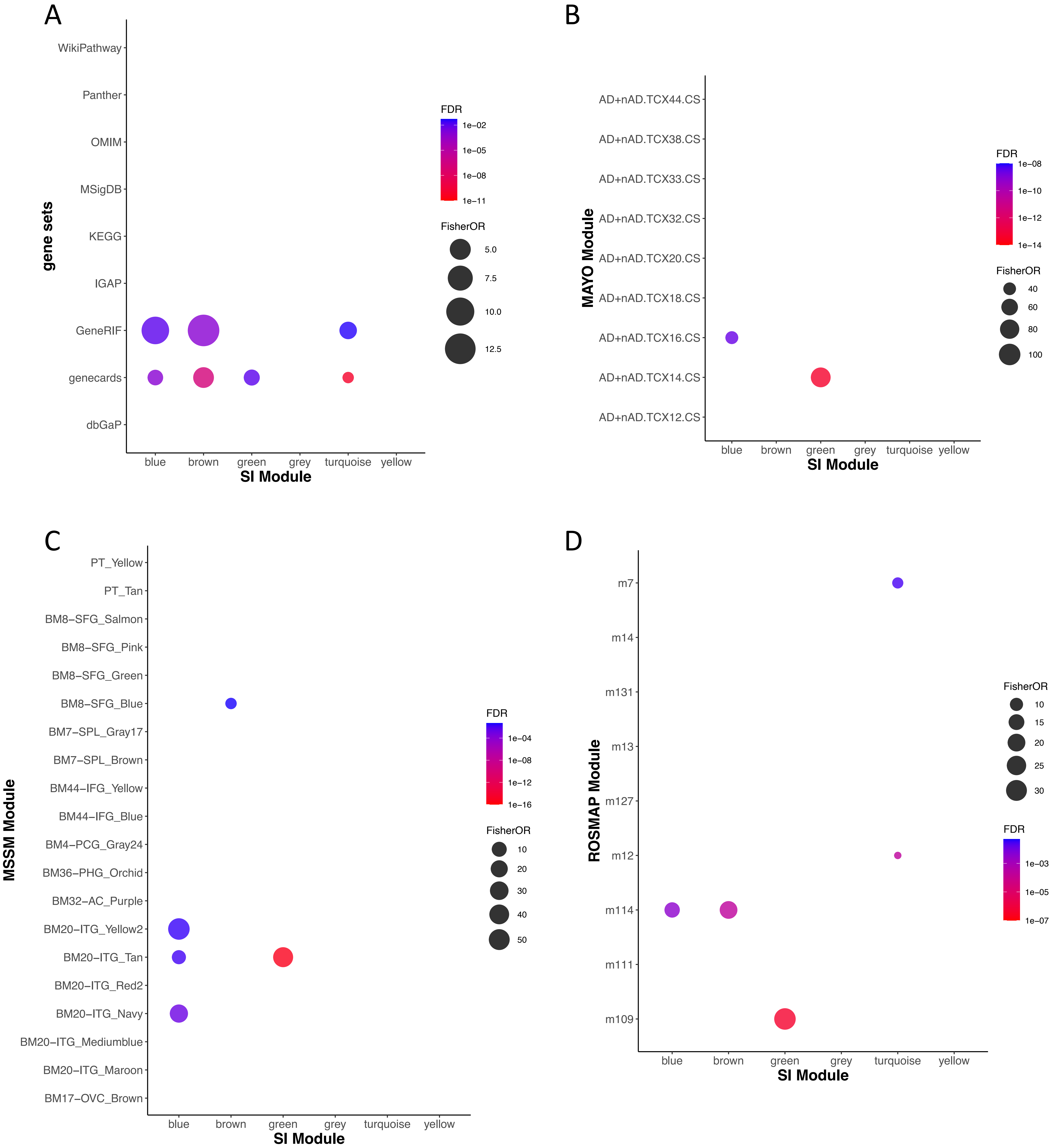

Supplementary Figure 5

A

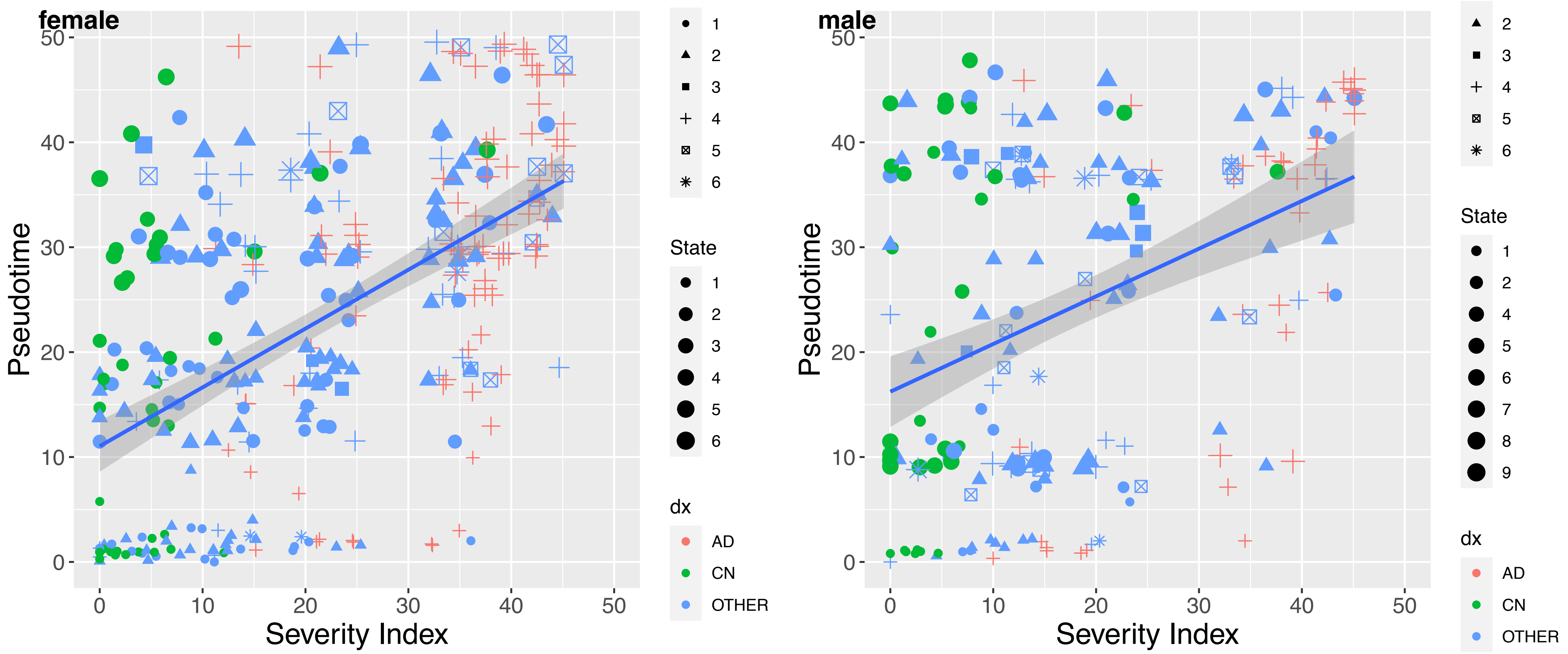

p < 2.2e-16, R^2 = 0.33

p = 5.5e-9, R^2 = 0.15

B

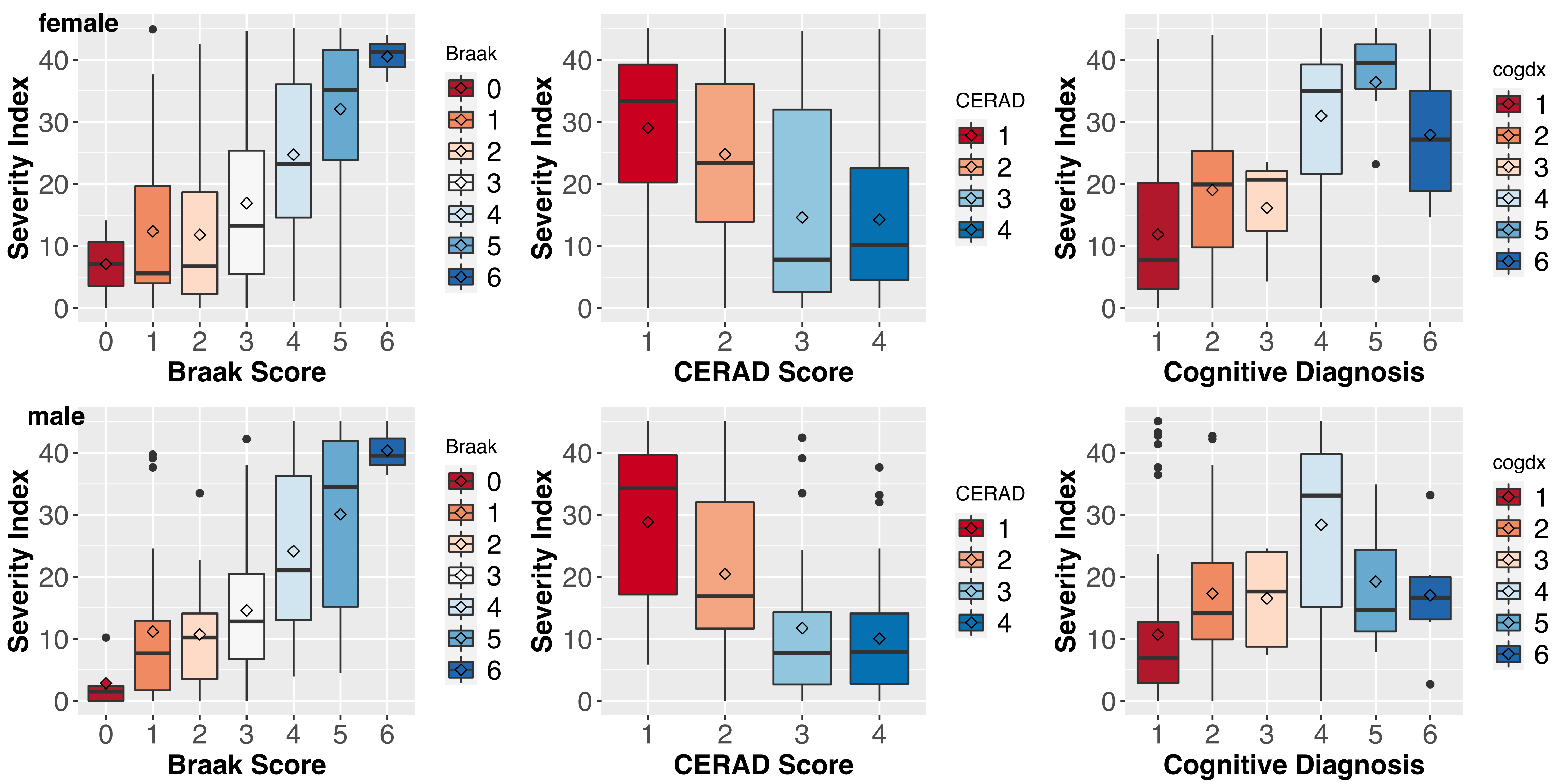

C

comparison	female				male			
	logistic		linear		logistic		linear	
	pseudotime	SI	pseudotime	SI	pseudotime	SI	pseudotime	SI
braak	1.21E-05	1.43E-17	3.01E-01	1.01E-06	2.63E-01	3.12E-13	2.44E-01	4.92E-07
CERAD	1.77E-05	1.48E-13	1.67E-03	2.63E-09	3.65E-02	8.87E-12	1.25E-01	1.50E-05
cogdx	4.26E-09	4.94E-23	-	-	3.17E-01	9.37E-09	-	-

Supplementary Figure 6

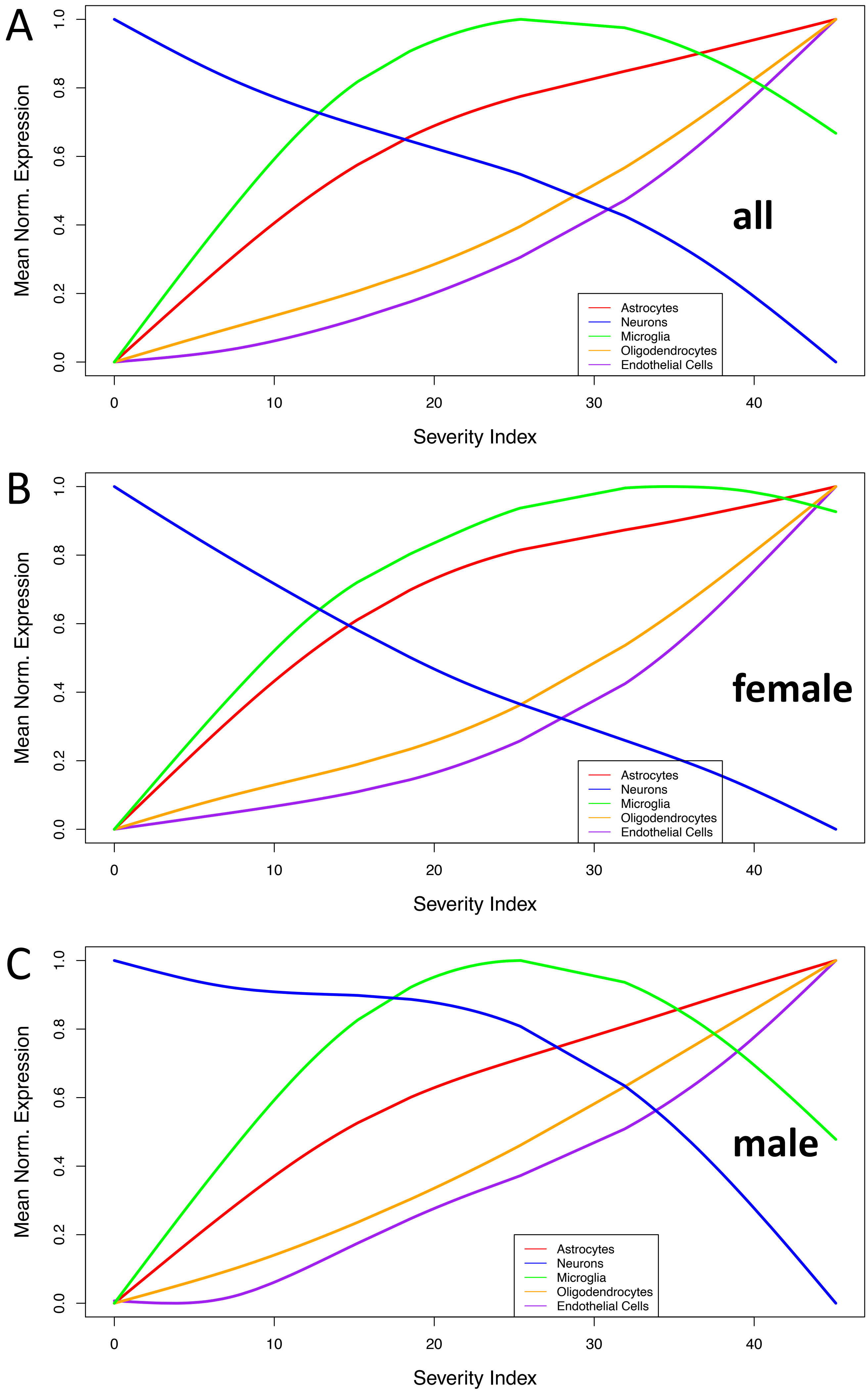

Supplementary Figure 7
